# Supplementary material for: Sustainable recovery of MBNL activity in autoregulatory feedback loop in myotonic dystrophy
Source: Mol Ther Nucleic Acids. 2022 Nov 3;30:438–48. doi: 10.1016/j.omtn.2022.10.023 (PMC9672890; doi:10.1016/j.omtn.2022.10.023)
Supplement: Document S1. Figures S1–S12 and Tables S1–S3 [file mmc1.pdf]

OMTN, Volume *30*

Supplemental information

Sustainable recovery of MBNL activity in autoregulatory feedback loop in myotonic dystrophy

Zuzanna Rogalska and Krzysztof Sobczak

Table S1. Primers used for genetic construct preparation.

name of primer	sequence 5` -> 3`
SRCglo_F	CCATTACAACCCGTGCAAGAGGGTAAGGAGT
SRCglo_R	AACTGAAAACATTGGATCTGGAAGGGGAAA
MB22mut_F	TGCTTGCTTGCTTGCTTCCAGTCAGGGTGGGCCG
MB22mut_R	GCAAGCAAGCAAGCAACGGCTCCAGGTGGAGCTGC
MB22del_F	CAAGGTCAGGGCTGCAGTGGGGGGGG
MB22del_R	GCAGCCCTGACCTTGGATGCGACGCG
GFPop_F	TAGGGATCCACCGGATCT
GFPop_R	CTTATCGTCGTCATCCTTGTAATCT
GFPvec_F	GATGACGACGATAAGGTGAGCAAGGGCGAGGAGC
GFPvec_R	TCCGGTGGATCCCTACTTGTACAGCTCGTCCATGCC

Table S2. siRNA sequences

siRNA	sequence 5` -> 3`
**siCtrl sense**	p- UCGAAGUAUUCCGCGUACGdTdT
**siCtrl antisense**	p- CGUACGCGGAAUACUUCGAdTdT
**siMBNL1 sense**	p- GGACGAGGUCAUUAGCCAUdTdT
**siMBNL1 antisense**	p- AUGGCUAAUGACCUCGUCCdTdT

Table S3. PCR primers used for alternative splicing assays and real-time qPCR analysis

PCR target	Primer set	sequence 5` -> 3`
MB22 ex22	pEGFP2_F	ACCGGACTCAGATCTCGAATG
	MBex3_R	ACCAGGCTTGGAGAAACAG
Minigene *Atp2a1* ex22	SrcMins_F	GATCTTCAAGCTCCGGGCCCTG
	SrcMins_R	AGCAATCAGCTAGTCAGTTGCC
Minigene *Nfix* ex7	pDUP51_F	GCAACCTCAAACAGACACCA
	pDUP51_R	AGCTTGTCACAGTGCAGCTC
Minigene *MBNL1* ex5	54ex_F	GCTGCCCAATACCAGGTCAAC
	pcDNA RT-spec_R	AAAGGACAGTGGGAGTGGC
INSR ex11	INSR_F	CCAAAGACAGACTCTCAGAT
	INSR_R	AACATCGCCAAGGGACCTGC
FLNB ex31	FLNB_F	GCTTCGGTGGTGTTGATATTC
	FLNB_R	GTCACTCACTGGGACATAGG
MYO5A ex22	MYO5A_F	GAACAACCGACAGCAGCAG
	MYO5A_R	TTACGGACCGTCTTATCCTG
MBNL2 ex5	MBNL2ex5_F	ATTTTCACCCTCCTGCACAC
	MBNL2ex5_R	CAAGACGCTGGGGTTAAAGA
MBNL2 ex7	MBNL2ex7_F	TCCTTTACCAAAGAGACAAGCAC
	MBNL2ex7_R	CTCAATGCAGATTCTTGGCATTCC
	MBNL1ex1_F	CAGCGACATGCAACAGTCTT

MBNL1 ex1	MBNL1ex1_R	TGTCAGCAGGATGAGCAAAC
NCOR2 ex19	NCOR2_F	ACACCCACAACCGGAATGAGCCTG
	NCOR2_R	GGACTTGGCTTTTCGGCTGCTG
PHKA1 ex19	PHKA1_F	TGCACACACTTGAGCTTCATGGA
	PHKA1_R	AAAGTCCACCTCCCCAGACTGGTC
GAPDH	GAPDH_F	GAGTCAACGGATTTGGTCGT
	GAPDH_R	TTGATTTTGGAGGGATCTCG
DMPK UTR	DMPK_F	GCGATCTCTGCCTGCTTACT
	DMPK_R	GTCCTAGGTGGGGACAGACA

a

gtgatgcggttttggcagtacatcaatgggcgtggatagcggtttgactcacggggatttccaagtctccaccccattgacgtcaatgggagtttgttttggcaccaaaa
tcaacgggactttccaaaatgtcgtaacaactccgccccattgacgcaaatgggcggtaggcgtgtacggtgggaggtctatataagcagagctggtttagtgaaccg
tcagatccgctagcgcaccggactcagatctcgaATGGCTGTTAGTGTCACACCAATTCGGGACACAAAATGGCTAACACTGGAAGTATGTA
GAGAGTTCCAGAGGGGGACTTGCTCACGGCCAGACACGGAATGTAAATTTGCACATCCTTCGAAAAGCTGCCAAGTTGAAA
ATGGACGAGTAATCGCCTGCTTTGATTCATTGAAAGGCCGTTGCTCCAGGGAGAACTGCAAATATCTTCATCCACCCCCACA
TTTAAAAACGCAGTTGGAGATAAATGGACGCAATAACTTGATTCAGCAGAAGAACATGGCCATGTTGGCCCAGCAAATGC
AACTAGCCAATGCCATGATGCCTGGTGCCCCATTACAACCCGTGCAAGAGGgtaaggagtgccctctctgtcccaagccctggccc
caccacagccccttccccatgacgccgcccccgccccgccccgtactttgcaggtggtaagtttctcagccctggcaggacctgtgtccgccccgttccccctgcgcct
gcaggggccacatctccggggcagccccactgcctcctcagcccccacagcccctatagcccccatgccacctccctgccttgataacagtgcctcttgtcctctctggccat
agGATAACTGTTCCCCCTCCTCCATCTCTGAGCCCGTGTCACAGgtatcacccccttcttgccctcagcccagctgctgtgcccctgccacccg
cgcccctcagccccttgcgcgtcgcatccaaggtcacttgtgctcgcagctccacctggagccgttgccactgctgctgctgcgcttccagtcagggtgggccgctggcctccc
actgggcgtcagtttggctcccaggccctgggcagtgccagcctctgggcccgtctgctgcgctgcgttgcgctggctgtgtgctgggctgtctttgctgtggggctgcagtggggg
ggggcggggtgtctggggacgcaggtgagtaggggagaaactggcagggtggtaagcttctgagcctccaggtaagtgcgtgcctgggagatgcacctgggagggacctcgctg
ccctcctgccgcctgcctcatcccttctctttccccttccagATCCAATGTTTTCAGTTGCACCAAGCTTAGCCACCAATGCATCAGCAGCCGCCTTTAAT
CCCTATCTGGGACCTGTTTCTCCAAGCCTGGTCCCGGCAGAGATCTTGCCGACTGCACCAATGTTGGTTACAGGGAATCCGG
GTGTCCCTGTACCTGCAGCTGCTGCAGCTGCTGCACAGAAATTAATGCGAACAGACAGACTTGAGGTATGTCGAGAGTACCA
ACGTGGCAATTGCAACCGAGGAGAAAATGATTGTCGGTTTGCTCATCCTGCTGACAGCACAATGATTGACACCAATGACA
ACACAGTCACTGTGTGTATGGATTACATCAAAGGGAGATGCTCTCGGGAAAAGTGCAAATACTTTCATCCCCCTGCACATT
TGCAAGCCAAGATCAAGGCTGCCCAATACCAGGTCAACCAGGCTGCAGCTGCACAGGCTGCAGCCACCGCAGCTGC
CATGGGAATTCCTCAAGCTGTACTTCCCCCATTACCAAAGAGGCCTGCTCTTGAAAAAAC
CAACGGTGCCACCGCAGTCTTTAACACTGGTATTTTCCAATACCAACAGGCTCTAGCCAACATGCAGTTACAACAGC
ATACAGCATTTCTCCCACCAGGCTCAATATTGTGCATGACACCCGCTACAAGTGTTGTTCCCATGGTGCACGGTGCTACGCC
AGCCACTGTGTCCGCAGCAACAACATCTGCCACAAGTGTTCCCTTCGCTGCAACAGCCACAGCCAACCAGATACCCATAATATCTGCCGA
ACATCTGACTAGCCACAAGTATGTTACCCAGATGGGAGATTACAAGGATGACGACGATAAGTAG
ggatccaccggatctagataactgatcataatcagccataccacatttgtagaggttttacttgctttaaaaaacctcccacacctccccctgaacctgaaacataaaatgaatgcaattgtt
gttgttaacttgtttattgcagcttataatggttacaaaaaagcaatagcatcacaaatttcacaaataaagcatttttttcactgcattctagttgtggtttgtccaaactcatcaatgtatctta

b

gtgatgcggttttggcagtacatcaatgggcgtggatagcggtttgactcacggggatttccaagtctccaccccattgacgtcaatgggagtttgttttggcaccaaaa
tcaacgggactttccaaaatgtcgtaacaactccgccccattgacgcaaatgggcggtaggcgtgtacggtgggaggtctatataagcagagctggtttagtgaaccg
tcagatccgctagcgcaccggactcagatctcgaATGGCTGTTAGTGTCACACCAATTCGGGACACAAAATGGCTAACACTGGAAGTATGTA
GAGAGTTCCAGAGGGGGACTTGCTCACGGCCAGACACGGAATGTAAATTTGCACATCCTTCGAAAAGCTGCCAAGTTGAAA
ATGGACGAGTAATCGCCTGCTTTGATTCATTGAAAGGCCGTTGCTCCAGGGAGAACTGCAAATATCTTCATCCACCCCCACA
TTTAAAAACGCAGTTGGAGATAAATGGACGCAATAACTTGATTCAGCAGAAGAACATGGCCATGTTGGCCCAGCAAATGC
AACTAGCCAATGCCATGATGCCTGGTGCCCCATTACAACCCGTGCAAGAGGgtaaggagtgccctctctgtcccaagccctggccc
caccacagccccttccccatgacgccgcccccgccccgccccgtactttgcaggtggtaagtttctcagccctggcaggacctgtgtccgccccgttccccctgcgcct
gcaggggccacatctccggggcagccccactgcctcctcagcccccacagcccctatagcccccatgccacctccctgccttgataacagtgcctcttgtcctctctggccat
agGATAACTGTTCCCCCTCCTCCATCTCTGAGCCCGTGTCACAGgtatcacccccttcttgccctcagcccagctgctgtgcccctgccacccg
cgcccctcagccccttgcgcgtcgcatccaaggtcacttgtgctcgcagctccacctggagccgttgcttgcttgcttccagtcagggtgggccgctggcctccc
actgggcgtcagtttggctcccaggccctgggcagtgccagcctctgggcccgtctgctgcgctgcgttgcgctggctgtgtgctgggctgtctttgctgtggggctgcagtggggg
ggggcggggtgtctggggacgcaggtgagtaggggagaaactggcagggtggtaagcttctgagcctccaggtaagtgcgtgcctgggagatgcacctgggagggacctcgctg
ccctcctgccgcctgcctcatcccttctctttccccttccagATCCAATGTTTTCAGTTGCACCAAGCTTAGCCACCAATGCATCAGCAGCCGCCTTTAAT
CCCTATCTGGGACCTGTTTCTCCAAGCCTGGTCCCGGCAGAGATCTTGCCGACTGCACCAATGTTGGTTACAGGGAATCCGG
GTGTCCCTGTACCTGCAGCTGCTGCAGCTGCTGCACAGAAATTAATGCGAACAGACAGACTTGAGGTATGTCGAGAGTACCA
ACGTGGCAATTGCAACCGAGGAGAAAATGATTGTCGGTTTGCTCATCCTGCTGACAGCACAATGATTGACACCAATGACA
ACACAGTCACTGTGTGTATGGATTACATCAAAGGGAGATGCTCTCGGGAAAAGTGCAAATACTTTCATCCCCCTGCACATT
TGCAAGCCAAGATCAAGGCTGCCCAATACCAGGTCAACCAGGCTGCAGCTGCACAGGCTGCAGCCACCGCAGCTGC
CATGGGAATTCCTCAAGCTGTACTTCCCCCATTACCAAAGAGGCCTGCTCTTGAAAAAAC
CAACGGTGCCACCGCAGTCTTTAACACTGGTATTTTCCAATACCAACAGGCTCTAGCCAACATGCAGTTACAACAGC
ATACAGCATTTCTCCCACCAGGCTCAATATTGTGCATGACACCCGCTACAAGTGTTGTTCCCATGGTGCACGGTGCTACGCC
AGCCACTGTGTCCGCAGCAACAACATCTGCCACAAGTGTTCCCTTCGCTGCAACAGCCACAGCCAACCAGATACCCATAATATCTGCCGA
ACATCTGACTAGCCACAAGTATGTTACCCAGATGGGAGATTACAAGGATGACGACGATAAGTAG
ggatccaccggatctagataactgatcataatcagccataccacatttgtagaggttttacttgctttaaaaaacctcccacacctccccctgaacctgaaacataaaatgaatgcaattgtt
gttgttaacttgtttattgcagcttataatggttacaaaaaagcaatagcatcacaaatttcacaaataaagcatttttttcactgcattctagttgtggtttgtccaaactcatcaatgtatctta

c

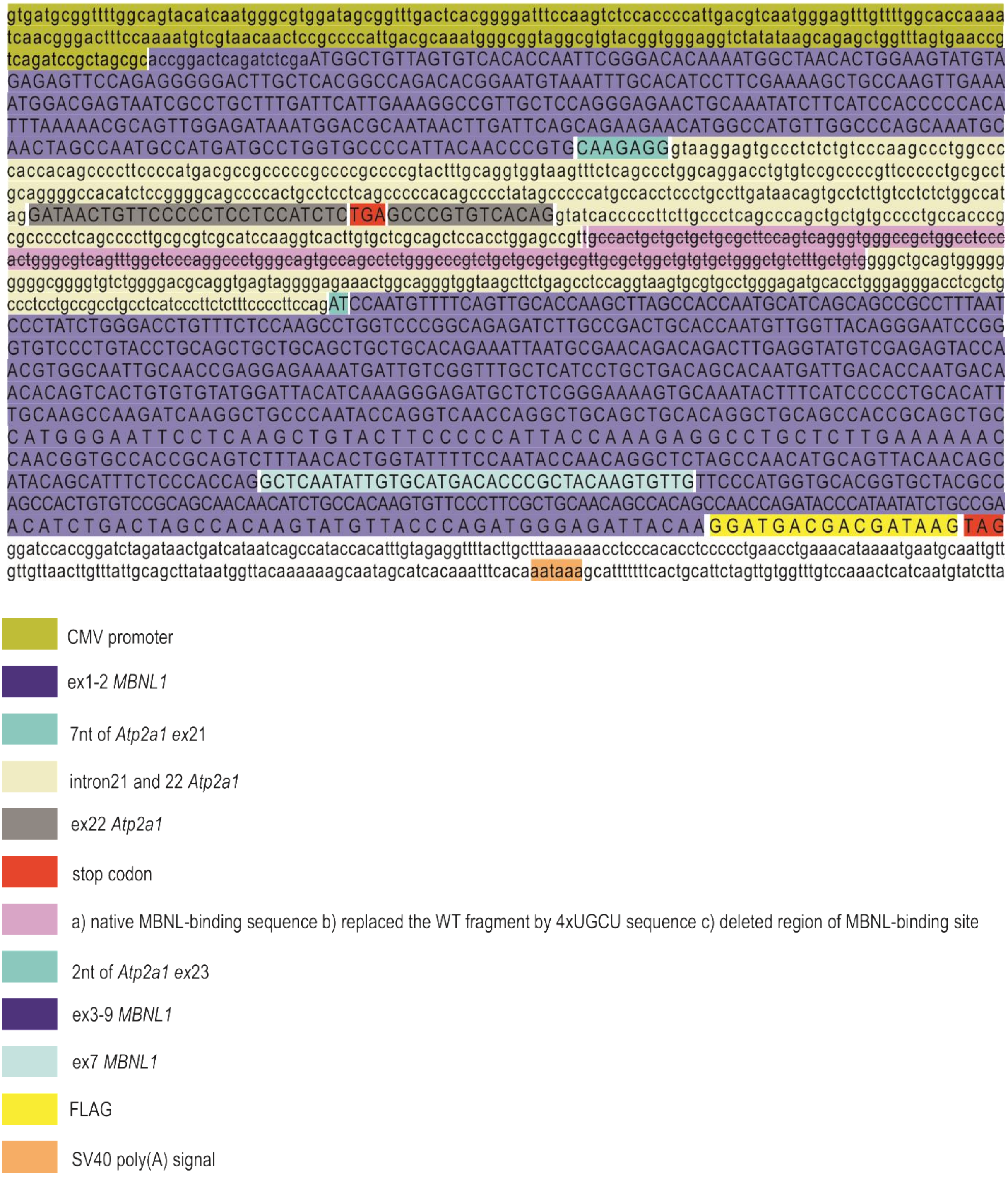

Figure S1. The sequences of MB22 construct with a) native (MB22#1) b) replaced the WT fragment by 4xUGCU sequence (MB22#2) or c) removed MBNL-binding motif (MB22-del) (related to Fig. 1a).

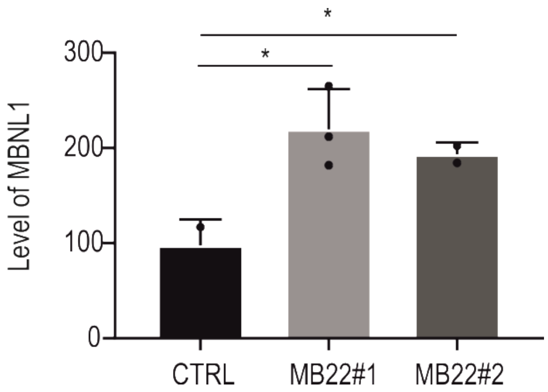

Figure S2. Total level of MBNL1 in MB22 treated cells (related to Fig. 1d).

Results of western blot analysis showing the level of total pool of MBNL1 in cells co-transfected with either MB22#1 or MB22#2 or GFP (CTRL). Anti-MBNL1 antibody staining was carried out. Bars represent average signal from n=3 independent experiments for each group normalized to mCherry. Co-transfection with mCherry expression vector was utilized as a normalization control of transfection. The MB22 treated cells were compared to GFP plasmid treated cells (CTRL); unpaired Student's *t-test*; *$P < 0.05$.

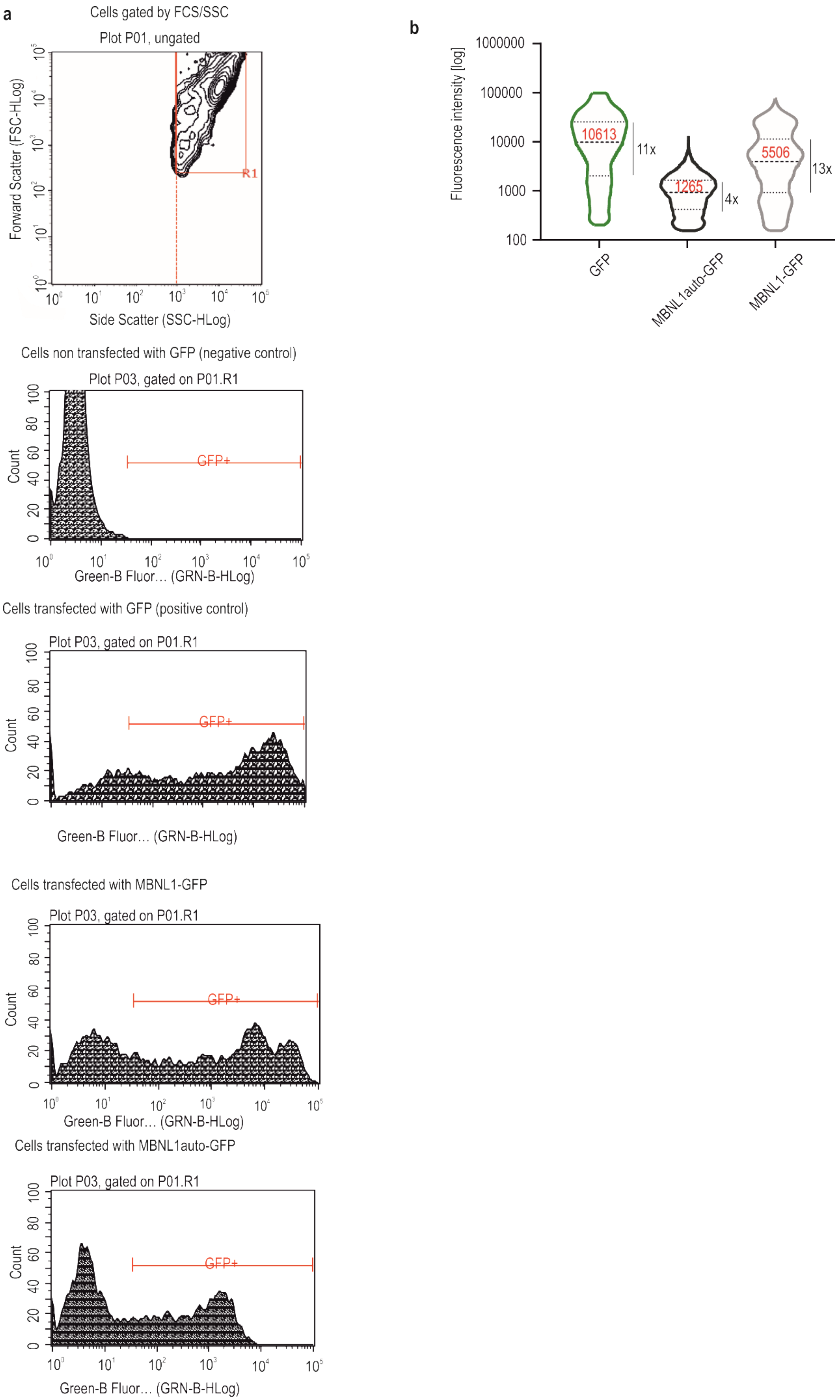
a
Cells gated by FCS/SSC
Plot P01, ungated
Forward Scatter (FSC-HLog)
Side Scatter (SSC-HLog)
R1
Cells non transfected with GFP (negative control)
Plot P03, gated on P01.R1
Count
GFP+
Green-B Fluor... (GRN-B-HLog)
Cells transfected with GFP (positive control)
Plot P03, gated on P01.R1
Count
GFP+
Green-B Fluor... (GRN-B-HLog)
Cells transfected with MBNL1-GFP
Plot P03, gated on P01.R1
Count
GFP+
Green-B Fluor... (GRN-B-HLog)
Cells transfected with MBNL1auto-GFP
Plot P03, gated on P01.R1
Count
GFP+
Green-B Fluor... (GRN-B-HLog)
b
Fluorescence intensity [log]
1000000
100000
10000
1000
100
10613
11x
1265
4x
5506
13x
GFP
MBNL1auto-GFP
MBNL1-GFP

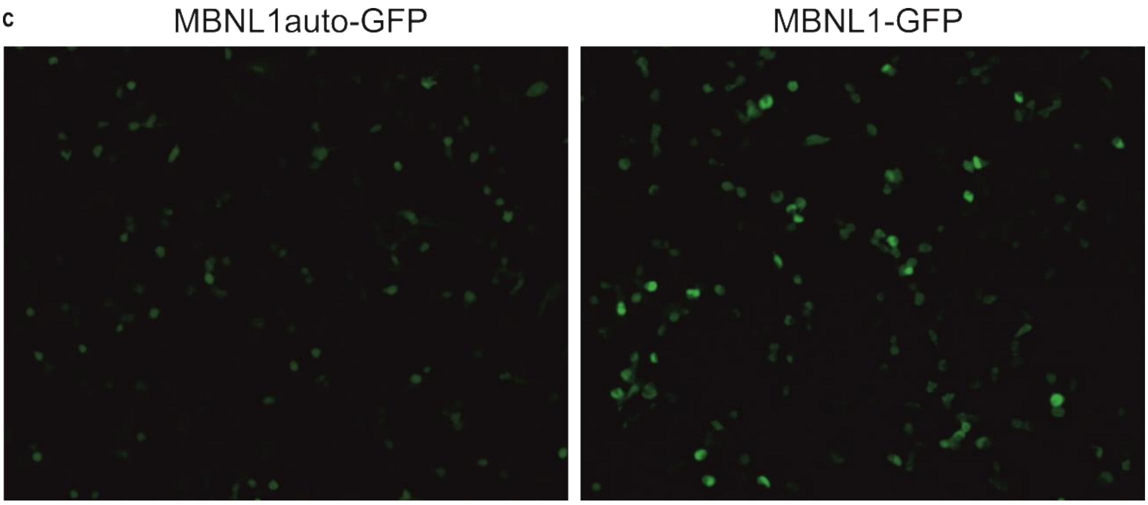

Figure S3. Representative flow cytometry gating and distribution of cells with different fluorescent signal (related to Fig. 3d).

a) For flow cytometry data analysis cells were first gated by Forward Scatter and Side Scatter (FSC/SSC). To distinguish between cells positive and negative for GFP channel, control cell sample (cells non-transfected with GFP referred as mock) were analyzed and utilized as background. Cells transfected with pEGFP-C1 plasmid with strong expression of GFP were also analyzed as positive control. Cells transfected with MBNL1-GFP and MB22#2-GFP represent fluorescence signal from MBNL1-GFP and MBNL1auto-GFP, respectively.

b) Violin plots showing the distribution of cells with different fluorescent signal of either GFP, MBNL1auto-GFP or MBNL1-GFP proteins. COS7 cells were transfected with adequate vectors 48 h prior to flow cytometry analysis. Median fluorescent intensity (black solid line) and 25th and 75th percentile of signal (dashed line) are shown. Fold-change between 25th and 75th percentile of signal for all analyses is also indicated. Cells with signal below 200 were rejected from analyses based on results for control experiment for mock-transfected cells. Signals below 200 were rejected based on analysis of non-transfected cells; Graphs represent values from n=4 independent biological replicates for each experimental condition; N=12916 (GFP), N=6781 (MBNL1auto-GFP) and N=10496 (MBNL1-GFP) cells. The obtained results from GFP, MBNL1-GFP and MBNL1auto-GFP were compared with mock treated as a control; Unpaired Student's *t-test*; *$P < 0.05$; ** $P < 0.01$; *** $P < 0.001$; ns, non-significant.

c) Representative confocal microscopy images showing localization of MBNL1auto-GFP and MBNL1-GFP in COS7 cells transfected with either autoregulated MB22#2-GFP or conventional MBNL1-GFP constructs, respectively.

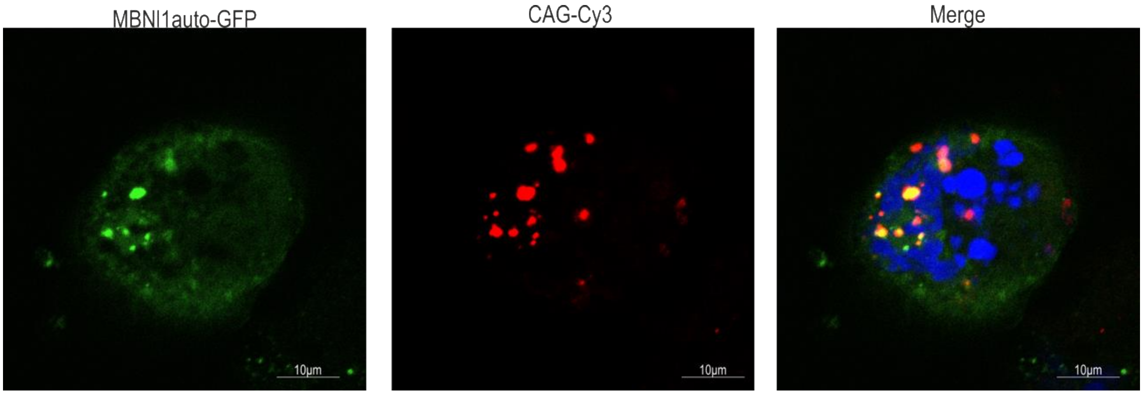

Figure S4. MBNL1auto colocalize with CUG^{exp} in MB22 treated cells (related to Fig. 4a).

Representative confocal images of FISH analysis to visualize the CUG^{exp} nuclear foci containing MBNL1auto in cells co-transfected with plasmid expressing mutant *DMPK* fragment containing CUG_{960} with MB22#2-GFP construct. RNA FISH was performed with DNA/LNA probes $(CAG)_6$-CA labeled at the 5′-end with Cy3 (CAG-Cy3) and MBNL1auto-GFP was visualized with GFP-specific filter (MBNL1auto-GFP); scale bar, 10µm.

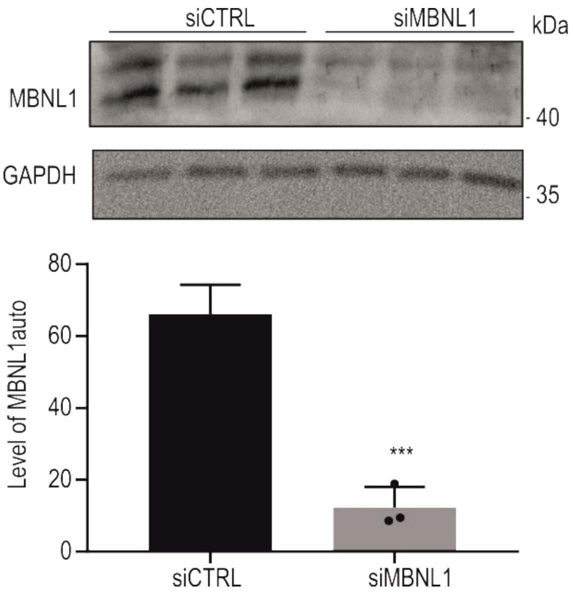

Figure S5. Confirmation of efficient knockdown of *MBNl1* (related to Fig. 4b).

Results of western blot analysis showing the level MBNL1 in COS7 cells treated with si*MBNL1* or siCTRL. Quantification of protein level (anti-MBNL1 antibody) is normalized to GAPDH. The results are averages from n=3 independent experiments; unpaired Student's *t-test*; *** $P < 0.001$ for comparison of siCTRL vs si*MBNL1* groups.

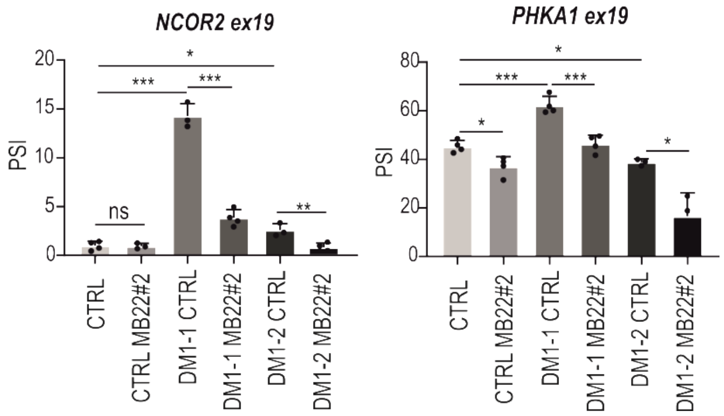

Figure S6. Correction of pathogenic mis-splicing in DM1 cells treated with MB22 lentiviruses (related to Fig. 5b).

Results of RT-PCR analyses showing changes in the regulation of two negatively regulated MBNL-dependent exons. Splicing changes are expressed as PSI. Three different cell line: fibroblast from healthy individuals (non-DM), and two DM1 patients (DM1-1 and DM1-2) were treated with control lentiviral vector (CTRL) or with lentivirus containing MB22#2-GFP sequence. Bars represent average from four independent experiments (dots); unpaired Student's *t-test*; *$P < 0.05$; ** $P < 0.01$; *** $P < 0.001$; ns, non-significant.

Supplementary files

a

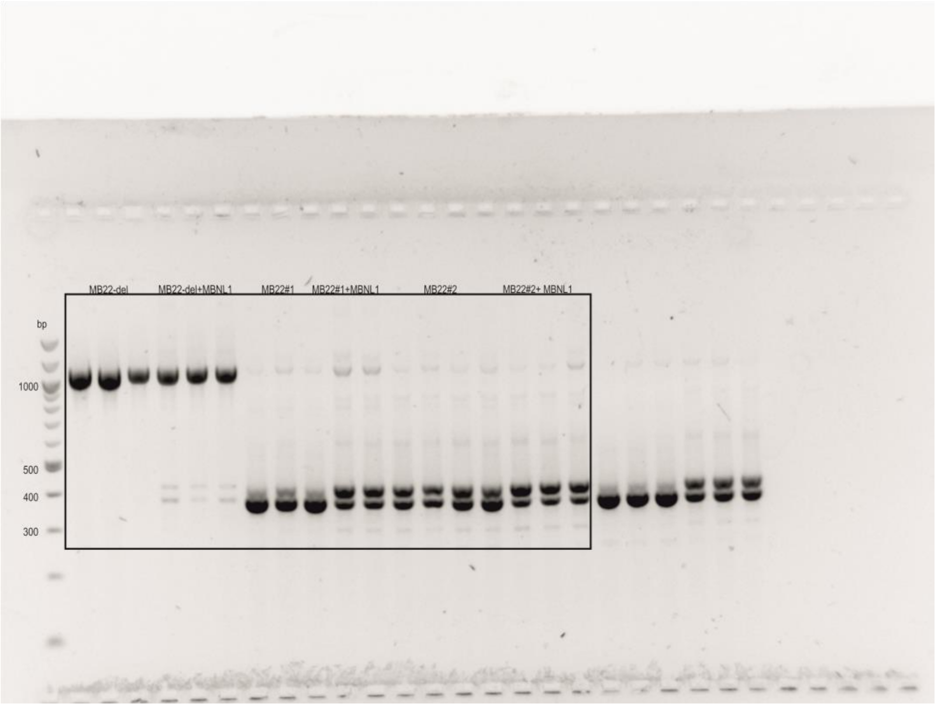

b

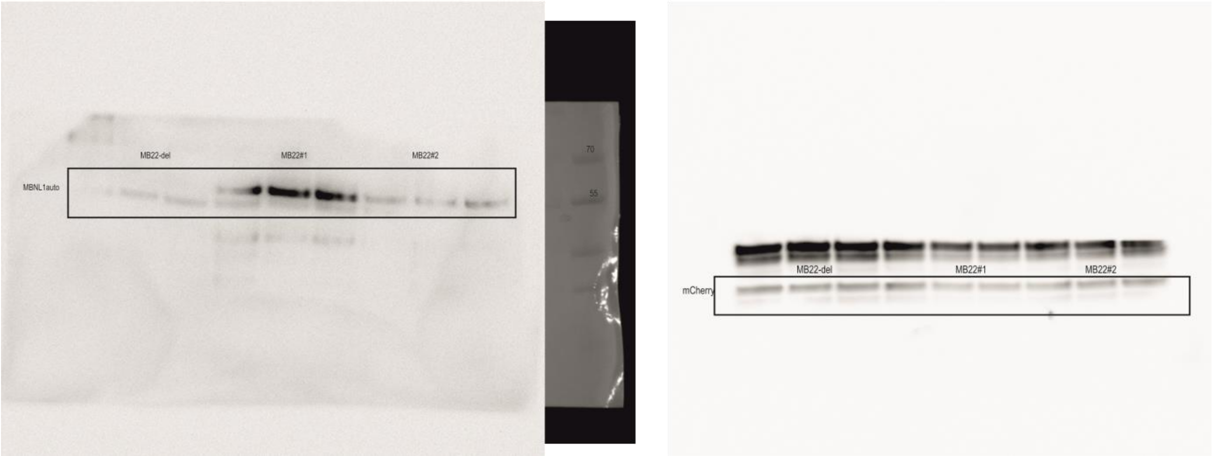

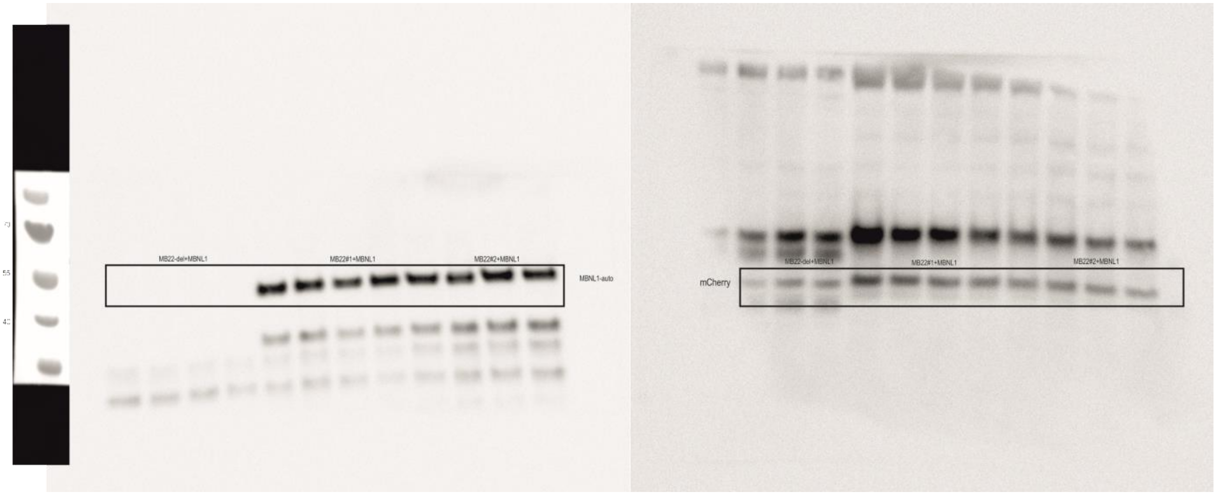

c

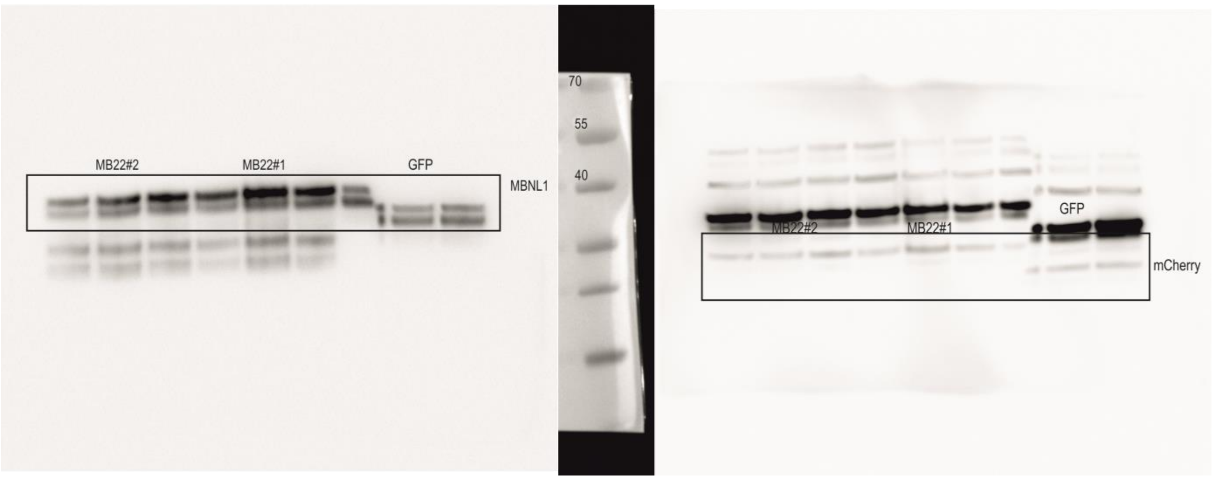

Figure S7 (related to Fig. 1c, d and Fig. S2).

a) Full agarose gel image for RT-PCR products results show in Fig. 1c. Fragments presented in Fig. 1c are indicated by black rectangles. **b)** Full blot image for western blot results show in Fig. 1d. Fragments presented in Fig. 1d are indicated by black rectangles. **c)** Full blot image for western blot results show in Fig. S2. Fragments presented in Fig. S2 are indicated by black rectangles.

COS7

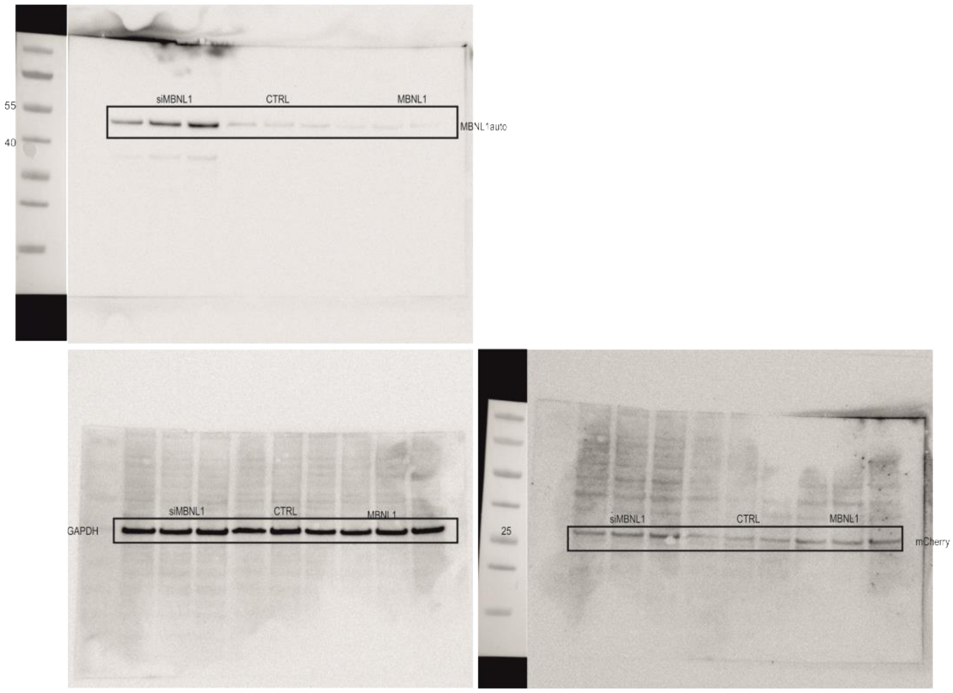

siMBNL1
CTRL
MBNL1
55
40
MBNL1auto
GAPDH
25
mCherry

HEK293

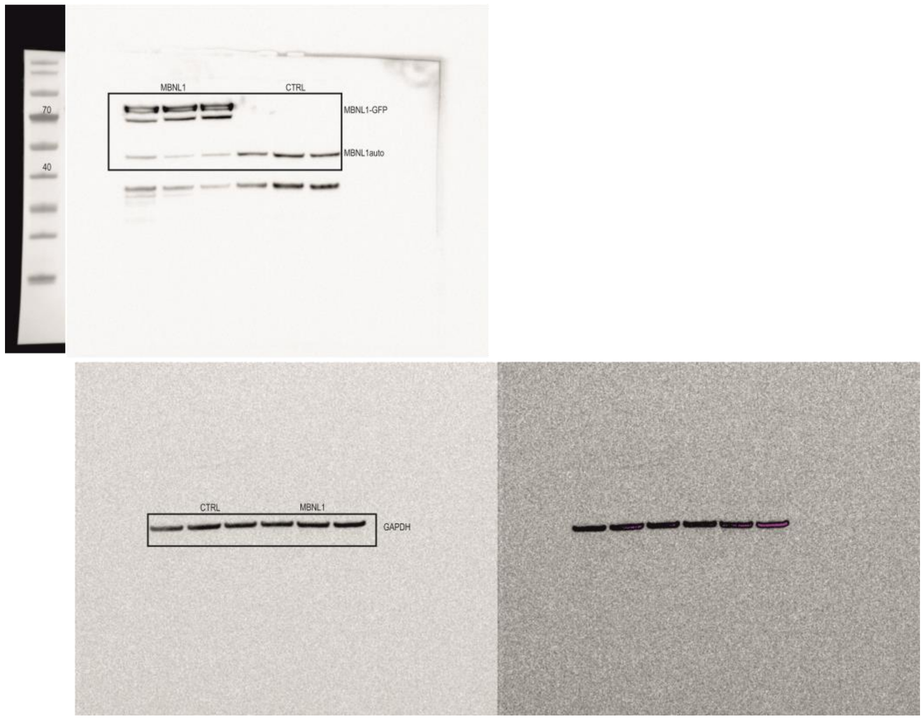

MBNL1
CTRL
70
40
MBNL1-GFP
MBNL1auto
GAPDH

MEF 1&2KO

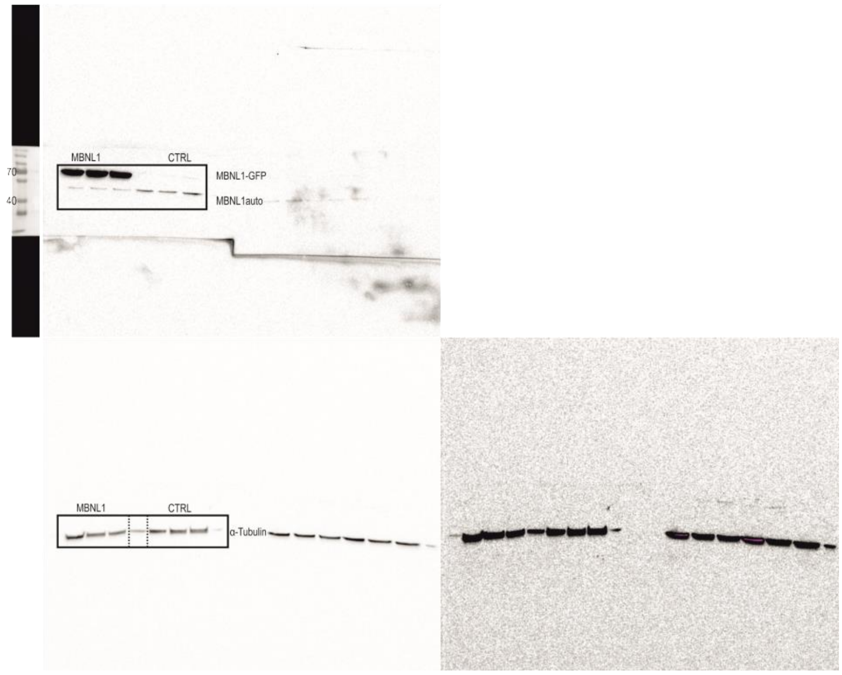

Figure S8 (related to Fig. 2).

Full blot images for western blot results show in Fig. 2. Fragments presented in Fig. 2 are indicated by black rectangles. Additional full blot image with GAPDH for HEK239 and α-Tubulin MEF 1&2KO was added with adjustment of contrast to see blot boundaries.

a

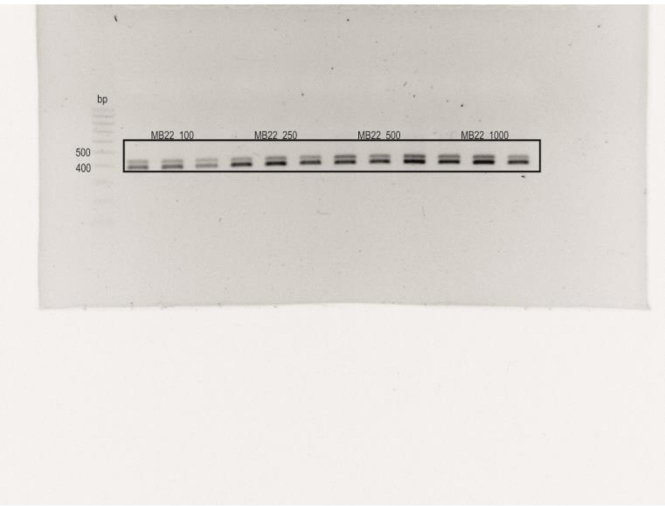

b

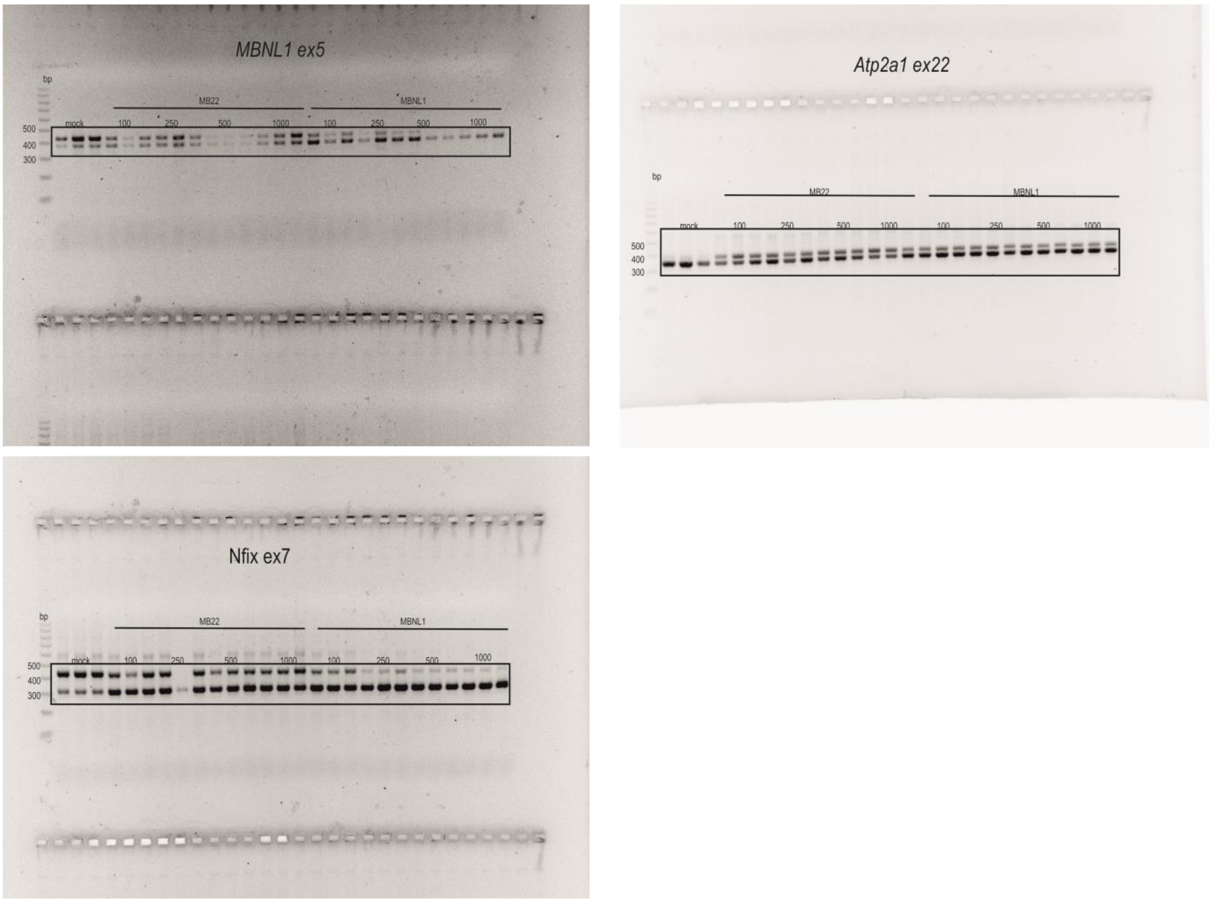

Figure S9 (related to Fig. 3a, b).

a) Full agarose gel image for RT-PCR results show in Fig. 3a. Fragments presented in Fig. 3a are indicated by black rectangles. **b)** Full agarose gel image for RT-PCR results show in Fig. 3b. Fragments presented in Fig. 3b are indicated by black rectangles.

a

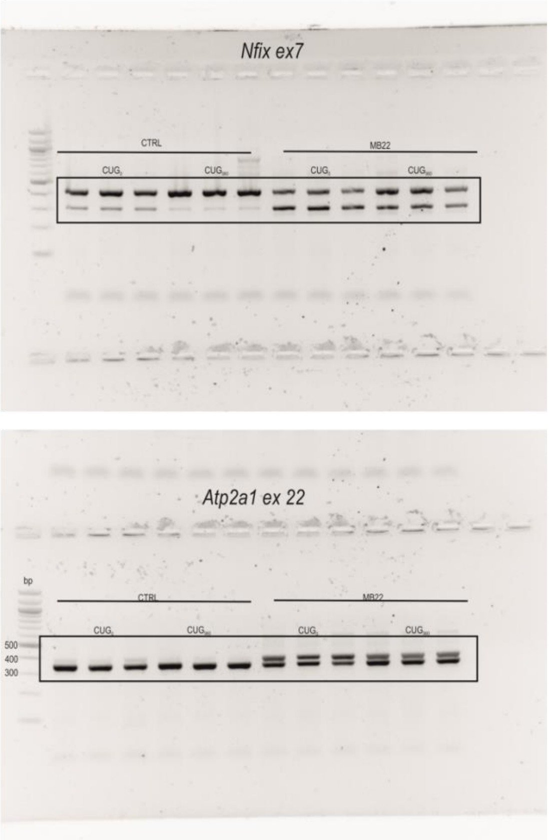

b

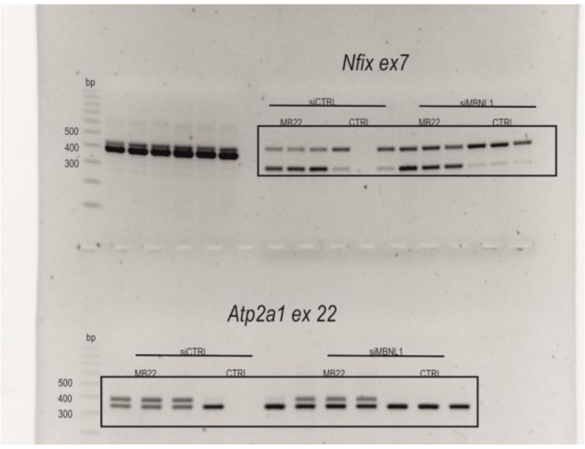

Figure S10 (related to Fig. 4b, c).

a) Full agarose gel image for RT-PCR results show in Fig. 4b. Fragments presented in Fig. 4b are indicated by black rectangles. **b)** Full agarose gel image for RT-PCR results show in Fig. 4c. Fragments presented in Fig. 4c are indicated by black rectangles.

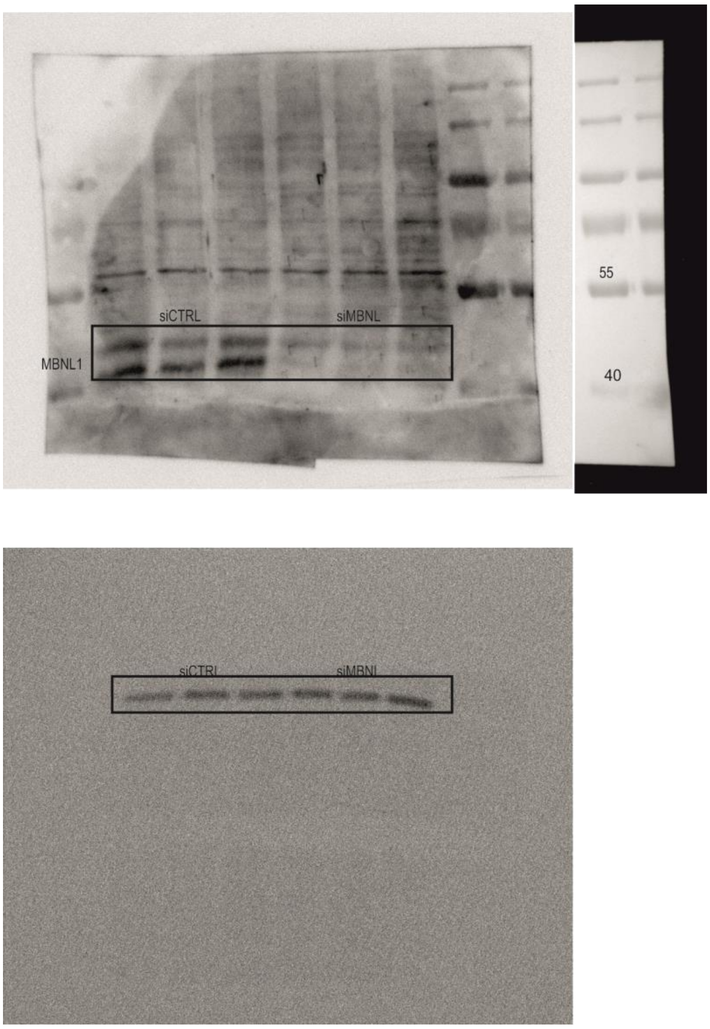

Figure S11 (related to Fig. S5).

Full blot images for western blot results show in Fig. S5. Fragments presented in Fig. S5 are indicated by black rectangles.

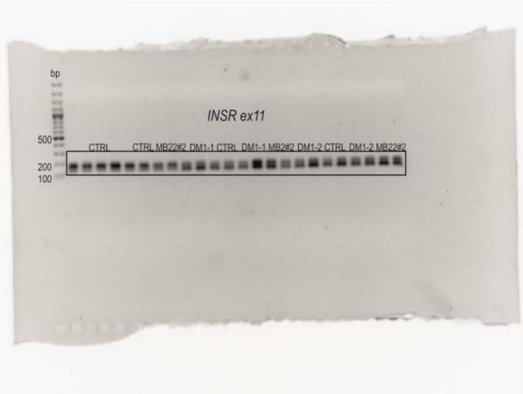
INSR ex11
bp
500
200
100
CTRL CTRL MB22#2 DM1-1 CTRL DM1-1 MB2#2 DM1-2 CTRL DM1-2 MB22#2

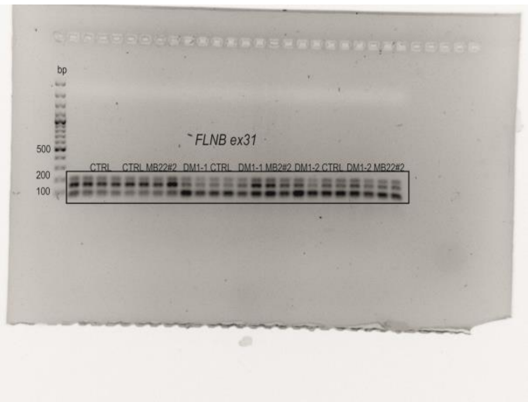
FLNB ex31
bp
500
200
100
CTRL CTRL MB22#2 DM1-1 CTRL DM1-1 MB2#2 DM1-2 CTRL DM1-2 MB22#2

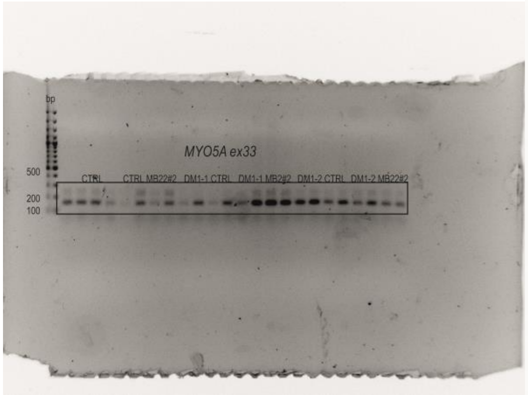
MYO5A ex33
bp
500
200
100
CTRL CTRL MB22#2 DM1-1 CTRL DM1-1 MB2#2 DM1-2 CTRL DM1-2 MB22#2

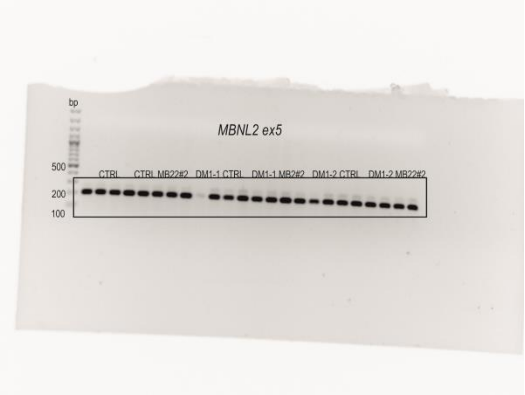
MBNL2 ex5
bp
500
200
100
CTRL CTRL MB22#2 DM1-1 CTRL DM1-1 MB2#2 DM1-2 CTRL DM1-2 MB22#2

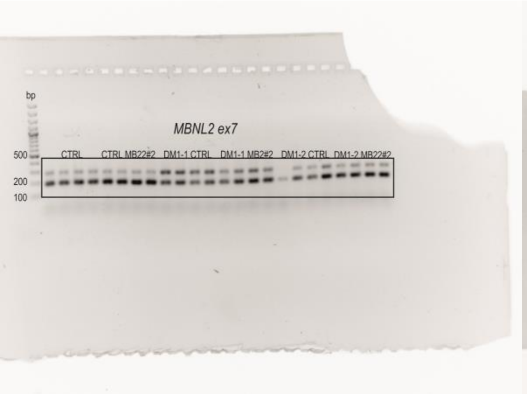
MBNL2 ex7
bp
500
200
100
CTRL CTRL MB22#2 DM1-1 CTRL DM1-1 MB2#2 DM1-2 CTRL DM1-2 MB22#2

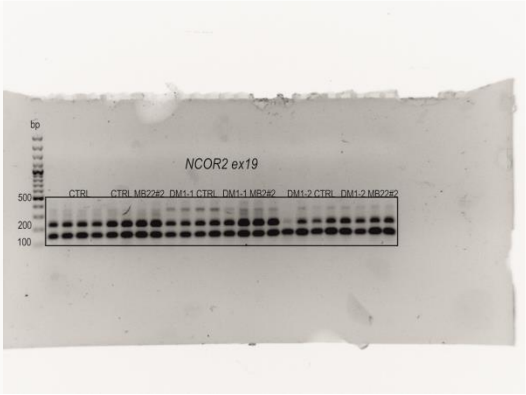
NCOR2 ex19
bp
500
200
100
CTRL CTRL MB22#2 DM1-1 CTRL DM1-1 MB2#2 DM1-2 CTRL DM1-2 MB22#2

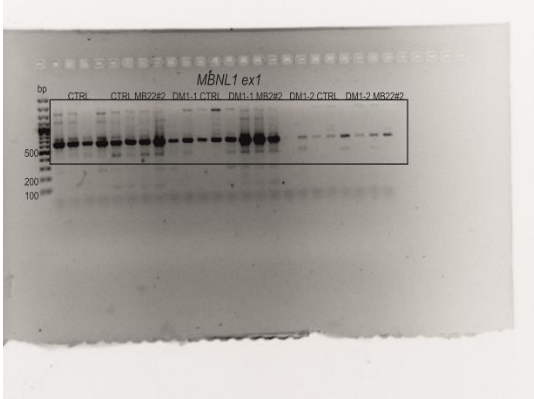
MBNL1 ex1
bp
500
200
100
CTRL CTRL MB22#2 DM1-1 CTRL DM1-1 MB2#2 DM1-2 CTRL DM1-2 MB22#2

Figure S12 (related to Fig. 5b and Fig. S6).

Full agarose gel image for RT-PCR results show in Fig. 5b and Fig. S6. Fragments presented in Fig. 5a and Fig. S6 are indicated by black rectangles.
