# Supplementary material for: Sustainable recovery of MBNL activity in autoregulatory feedback loop in myotonic dystrophy
Source: Mol Ther Nucleic Acids. 2022 Nov 3;30:438–48. doi: 10.1016/j.omtn.2022.10.023 (PMC9672890; doi:10.1016/j.omtn.2022.10.023)
Supplement: Document S3. Article plus supplemental information [file mmc2.pdf]

Molecular Therapy
Nucleic Acids
Original Article

Sustainable recovery of MBNL activity in autoregulatory feedback loop in myotonic dystrophy

Zuzanna Rogalska[1] and Krzysztof Sobczak[1]

[1]Department of Gene Expression, Institute of Molecular Biology and Biotechnology, Adam Mickiewicz University, Uniwersytetu Poznanskiego 6, 61-614 Poznan, Poland

Muscleblind-like proteins (MBNLs) are RNA-binding proteins essential for the developmental regulation of various processes including alternative splicing. Their activity is misregulated in myotonic dystrophy type 1 (DM1), an incurable genetic, neuromuscular disorder caused by uncontrolled expansion of CTG repeats. Mutant RNAs containing hundreds or thousands of repeats efficiently sequester MBNL proteins. As a consequence, global alternative splicing abnormalities are induced. Importantly, the size of expansion differs significantly not only between patients but also between different parts of the same muscle as a consequence of somatic expansion. One of the potential therapeutic strategies in DM is overexpression of MBNLs. However, gene therapy tools might induce excessive activity of MBNLs, what in turn might change the metabolism of many RNAs. To overcome these limitations, we designed an autoregulated MBNL1 overexpression system. The genetic construct contains an MBNL1-coding sequence separated by the fragment of *ATP2A1* pre-mRNA with an MBNL-sensitive alternative exon containing stop codon in the reading frame of MBNL1. Inclusion of this exon leads to the arrangement of an inactive form of the protein, but exclusion gives rise to fully active MBNL1. This approach enables the autoregulation of the amount of overexpressed MBNL1 with high dynamic range which ensures a homogeneous level of this protein in cells treated with the genetic construct. We demonstrated beneficial effects of an autoregulated construct on alternative splicing patterns in DM1 models and cells derived from patients with DM1.

INTRODUCTION

Three *Muscleblind*-like genes (*MBNLs*) encode for RNA-binding proteins essential for regulation of various processes of RNA metabolism including alternative splicing, polyadenylation, RNA localization, and stability.[1–3] MBNL1 is mainly expressed in skeletal muscles, MBNL2 in brain, and MBNL3 in placenta and during muscle cell differentiation, where they regulate hundreds of alternative splicing events leading to the expression of adult-specific mRNA isoforms.[4–6] All family members bind to RNA via four zinc fingers (ZFs) organized in two tandems connected by a long linker. MBNLs recognize their targets through closely organized multiple 5′-YGCY-3′ sequence motifs, where Y represents a pyrimidine.[7,8] The location of the MBNL-binding sites on target RNAs determines if the alternative exon is included or excluded. The presence of the preferred sequence motif upstream of or within the exon leads to the exon exclusion while its presence downstream facilitates exon inclusion.[3,8,9]

Activity of MBNLs is significantly misregulated in many diseases, including myotonic dystrophy (DM). DM1 and DM2 are autosomal-dominant genetic multisystemic disorders with symptoms mainly affecting skeletal and cardiac muscles and the central nervous system.[10] They are caused by uncontrolled expansion of either CTG in the 3′ untranslated region (3′ UTR) of the dystrophia myotonica protein kinase (*DMPK*) gene or CCTG repeats within the intron of the cellular nucleic-acid-binding protein (*CNBP*) gene, respectively.[11,12] RNA containing expanded CUG (CUG^{exp}) or CCUG ($CCUG^{exp}$) form thermodynamically stable long hairpin structures that efficiently bind and sequester MBNL proteins from nucleoplasm and, together, form nuclear foci.[13,14] Efficiency of sequestration depends on the length of CUG^{exp} and $CCUG^{exp}$, which is significantly variable in different patients, ranging from hundreds to thousands of repeats.[11] As a consequence, different severities of global abnormalities of alternative splicing and alternative polyadenylation are observed, which is further associated with disease severity and progression.[8,11,15,16] Generally, the activity of MBNLs in skeletal muscles and heart is significantly lowered to the level observed in newborns. Moreover, the length of CUG^{exp} in DM1 significantly differs in different tissues, which is a consequence of somatic repeat expansion occurring during the lifespan of patients.[17,18] More extensive somatic expansion is observed in heart and skeletal muscles than in blood and many other tissues.[18–20] The length of repeats can be different even in nuclei of the same muscle fiber, which reflects somatic mosaicism of CUG^{exp}.[21,22] Moreover, the length of $CCUG^{exp}$ may be also deeply heterogeneous in the same patient, as indicated by recent studies showing variability in repeat length and repeat composition.[23,24]

Received 18 March 2022; accepted 31 October 2022;
https://doi.org/10.1016/j.omtn.2022.10.023.

Correspondence: Krzysztof Sobczak, Department of Gene Expression, Institute of Molecular Biology and Biotechnology, Adam Mickiewicz University, Uniwersytetu Poznanskiego 6, 61-614 Poznan, Poland.
E-mail: ksobczak@amu.edu.pl

 © 2022 The Authors.
This is an open access article under the CC BY license (http://creativecommons.org/licenses/by/4.0/).

Hence, the sequestration of MBNL proteins is highly heterogeneous in both DM forms, even in the same tissue of the same patient.

So far, there are no cures for these diseases; however, a few therapeutic approaches were tested in cell and animal models of DM, and some of them demonstrated rescue of disease phenotype. The therapeutic strategies for DM1 may be grouped into three main categories: (1) induction of degradation of mutant RNA containing CUG^{exp}, (2) the release of MBNLs from pathogenic sequestration, and (3) the increase of MBNL expression. The second category involves the application of CUG^{exp}-specific small molecules or antisense oligonucleotides (ASOs), which can inhibit the formation of the pathogenic RNA-MBNL complex and lead to the improvement of alternative splicing defects.[25–28]

The third mentioned category involves either manipulation of an endogenous pool of MBNLs, e.g., the use of non-steroidal anti-inflammatory drugs that modify activity of *MBNL1* promoter,[29] or antagomiRs targeting *miR-23b* or *miR-218* to increase translation from *MBNL1* and *MBNL2* mRNAs,[28] or the application of gene therapy tools for MBNL overexpression.

Proof of concept for gene therapy was described for the DM1 mouse model, *HSA*-LR, expressing a transgene with 220 CTG repeats in 3′ UTR of human skeletal actin gene (*HSA*). The MBNL1-encoding transgene was delivered to the *HSA*-LR mouse via transduction with adeno-associated virus (AAV).[30] Twenty-three weeks after intramuscular AAV injection, the MBNL1 overexpression rescued the splicing defects of many MBNL-sensitive genes. At the same time, muscle hyperexcitability was missing. Otherwise, the normal structure of myofibers was not recovered, suggesting that the overexpression was insufficient to rescue this phenotype.[30] More recently, it was shown that truncated MBNL1 overexpressed from the AAV vector, which preserved both ZFs tandems but is deprived of the C-terminal domain, binds to the CUG^{exp} with high affinity, leading to the release endogenous MBNL proteins from sequestration and then rescue of disease phenotypes in the DM1 mouse model.[31] In other research, it was shown that the cross of *HSA*-LR with the mouse model with multisystemic overexpression of MBNL1 or overexpression restricted just to the skeletal muscles (*MBNL1*-OE) showed the rescue of DM-like defects, including the decrease of percentage of fibers with central nuclei reflecting myopathy changes. On the other hand, the long-term, multisystemic overexpression of MBNL1 in wild-type mice and another DM1 mouse model, DM200^{+}, which utilizes the doxycycline-dependent promoter that controls the expression of a transgene with 200 CTG repeats in the 3′ UTR of the *DMPK* gene, led to reduced body weight and increased mortality.[32,33] Moreover, intramuscular injection of AAV encoding the full length of MBNL1 to the wild-type (WT) mice induced formation of muscle fibers with internal nuclei, which indicates muscle damage.[31] These results suggest that there are some limitations in therapeutic strategies against DM based on the uncontrolled overexpression of MBNLs, especially considering the significant variability in the size and expression level of CUG^{exp} in different tissues of patients with DM.

To overcome the limitations caused by heterogeneity of CTG/CCTG repeat expansions and, consequently, different levels of MBNL insufficiency in different cells/myofibers, we designed and tested the autoregulated MBNL1 overexpression constructs, which enable the significant production of MBNL1 only if its level in the cell is too low and potentially can be controlled by heterogeneity of the CUG^{exp} load. We demonstrated that the level of the protein assembling from the construct is homogeneous from cell to cell, is controlled by a pool of available MBNLs, and has therapeutic potential to correct the alternative splicing abnormalities in cellular models of DM1. Considering DM1 and DM2 acquire the same mechanism of MBNL sequestration and, in consequence, share similar missplicing events, the potential therapeutics may be suitable for both.[34]

RESULTS

Constructs for autoregulated overexpression of MBNL1auto

For the autoregulated overexpression of MBNL1, we designed the hybrid genetic construct MB22#1, which contains an MBNL1-encoding sequence separated by a fragment of the *ATP2A1* gene containing MBNL-sensitive alternative exon 22 (ex22) and neighboring introns (Figures 1A and S1). We decided to choose ex22 because of its high sensitivity to MBNL regulation. In skeletal muscles, the isoform with the inclusion of ex22 predominates, but in affected muscles of patients with DM1, different levels of ex22 exclusion are observed (Figure 1B), which is correlated with disease severity.[35] The sequence of the *ATP2A1* gene fragment was incorporated between ex2 and ex3 of *MBNL1* so as to not disturb the structure of any of the ZF tandems. Alternative ex22 is positively regulated by all MBNL paralogs.[36] We hypothesized that the inclusion of ex22 may lead to the production of a truncated, inactive form of the protein because of the presence of in-frame stop codon (Figure 1A). Therefore, in cells with low levels of MBNLs, ex22 can be excluded during pre-mRNA maturation, and the fully active form of MBNL1 protein (MBNL1auto) can be assembled. We also designed two other constructs by replacing native MBNL-binding sequences in intron 22 with sequences showing different sensitivities to MBNLs.[37] First, MB22#2 contains the sequence with four consecutive 5′-UGCU motifs as an MBNL-binding site. Second, control MB22-del has a deletion of the native MBNL-binding site of *ATP2A1* (Figure 1A). Moreover, at the C terminus of the MBNL1auto sequence, either FLAG tag or GFP tag was added. We hypothesized that these constructs allow the adjustment of different amounts of MBNL1auto due to different efficiency of ex22 inclusion.

To test this hypothesis, we co-transfected COS7 cells with one of three generated MB22 constructs containing different MBNL-sensitive elements and either MBNL1-GFP (without [w/o] autoregulatory cassette) or as a control GFP expressing vector. The RT-PCR base splicing assay showed that the percentage of mRNA isoforms with the exclusion of ex22 depends on the presence of an MBNL-sensitive RNA regulatory element (MB22#1, -#2, or -del) and the level of the MBNL pool in cells. We decided to measure the ex22 exclusion, as this isoform can produce functional MBNL1auto. As expected, the exclusion rate of ex22 was higher for MB22#1 and MB22#2 constructs in cells with a basal pool of endogenous MBNLs and significantly

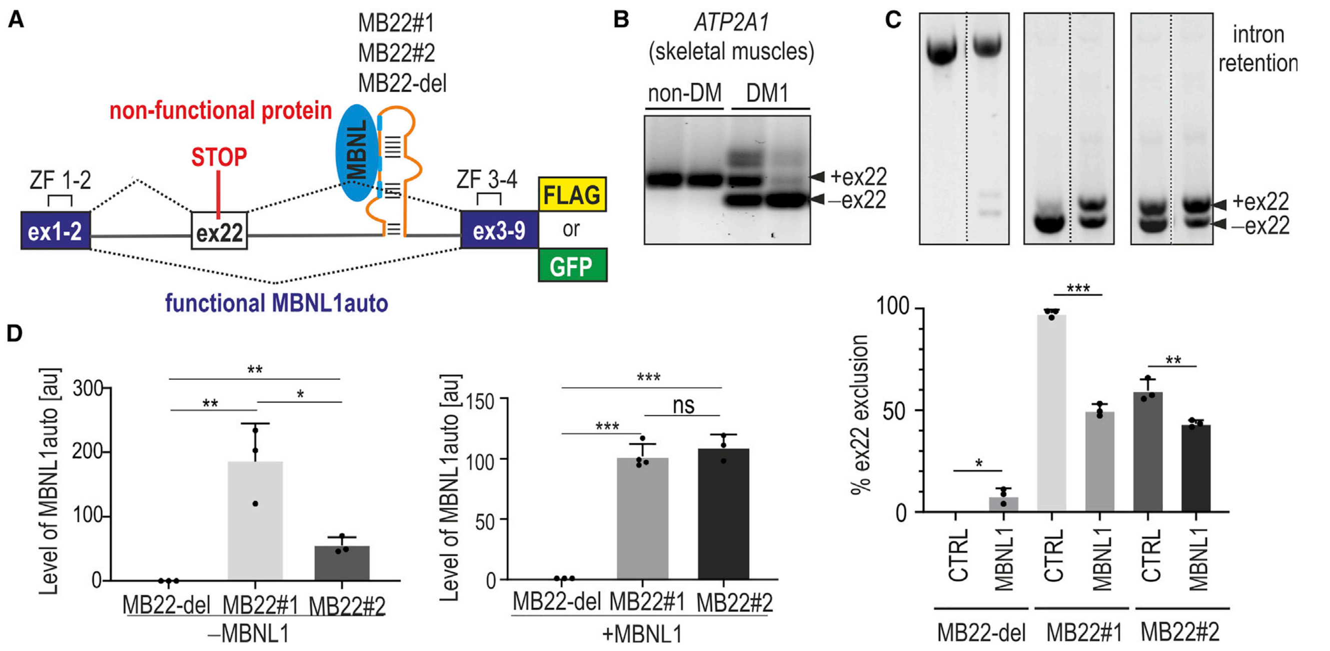

Figure 1. Inclusion of the alternative exon to mRNA encoding MBNL1auto depends on the MBNL-binding RNA regulatory motif

(A) The scheme of the genetic construct for autoregulated overexpression of MBNL1auto. The *MBNL1* coding sequence was divided into two parts separated by an intron21/exon (ex)22/intron22 sequence from human *ATP2A1*. Ex1-2 is a sequence of the first two exons of *MBNL1*, containing the first zinc finger tandem (ZF1–ZF2); ex3-9 is a sequence of cDNA of ex3–ex9 with ZF3 and ZF4. *ATP2A1* fragment contains the alternative ex22 flanked from both sides by introns and 7 bp of ex21 and 2 bp of ex23 to maintain correct splicing regulation and to keep the open reading frame of MBNL1auto. The inclusion of ex22, positively regulated by MBNLs, leads to premature translation termination and the arrangement of an inactive form of protein. The MB22 construct contains the wild-type sequence recognized by MBNLs (blue) within intron22 of *ATP2A1* (MB22#1). This sequence was replaced by 4xUGCU MBNL-binding motif (MB22#2) or completely removed (MB22-del; orange). GFP and FLAG are tags located in the frame of MBNL1auto. (B) Alternative splicing profile of *ATP2A1* ex22 in two normal adult skeletal muscles (non-DM) and two different skeletal muscles from patients with DM1 (DM1) analyzed by RT-PCR. Isoforms with and without ex22 are marked. (C) Results of RT-PCR analysis of ex22 exclusion in cells transfected with MB22 constructs containing different MBNL-sensitive elements (MB22#1, -#2, and -del) and with MBNL1-GFP (MBNL1) or GFP (CTRL) overexpression. The percentage of alternative ex22 exclusion reflects mRNA isoforms coding for the active form of the protein. Bars represent average from n = 3 independent experiments (dots) for each experimental condition with standard deviation (SD). (D) Results of western blot analysis showing the level of MBNL1auto in cells transfected with either MB22-del, -#1, or -#2 (left; –MBNL1) or co-transfected with these three constructs and MBNL1-GFP (right; +MBNL1). Anti-FLAG antibody staining was carried out as FLAG sequence is fused to the C-terminal end of MBNL1auto in each MB22 construct. Bars represent average signal (arbitrary units [a.u.]) from n = 3–4 independent experiments for each group normalized to mCherry. Co-transfection with mCherry expression vector was utilized as a normalization control of transfection. (B and C) Unpaired Student's t test; *p < 0.05; **p < 0.01; ***p < 0.001; ns, non-significant.

decreased with MBNL1-GFP overexpression (Figure 1C). Unexpectedly, the level of mRNA with excluded ex22 from the control MB22-del construct is very low because the isoform with retention of both introns predominates.

Next, we analyzed the level of MBNL1auto in cells transfected with one of three autoregulated MB22 constructs (Figure 1D, left graph) or co-transfected with MB22s and non-autoregulated MBNL1-GFP (Figure 1D, right graph) using western blot. A higher amount of protein was produced in cells treated with MB22#1. On the other hand, a similar amount of MBNL1auto protein was detected in cells co-transfected with either MB22#1 or MB22#2 and MBNL1-GFP. These results are consistent with a RT-PCR-base splicing assay showing the percentage of mRNA isoforms with exclusion of ex22 (Figure 1C). The highest ex22 exclusion was in cells treated with MB22#1, suggesting the highest potential to produce the full length of MBNL1auto. Furthermore, we evaluated the total pool of MBNL1 after MB22 overexpression. The western blot analysis showed about a 2-times increase of the MBNL1 pool compared with the endogenous level of this protein (Figure S2). MBNL1auto was not detected in cells transfected with MB22-del (Figure 1D), which corroborated the results of mRNA splicing analysis (Figure 1C).

All these experiments demonstrate that autoregulated MB22 constructs are capable of overexpressing different levels of MBNL1auto in cells with different pools of MBNL proteins.

The effect of a pool of MBNLs on production of MBNL1auto from the autoregulated MB22 construct

By design, the amount of produced MBNL1auto should also differ in cells with different endogenous MBNL pools. Therefore, we overexpressed MB22#1 in three cell lines with different levels of MBNL pools (total of MBNL1, -2, and -3): in the COS7 line and the HEK293 line, with relatively high and low endogenous pools of MBNLs,

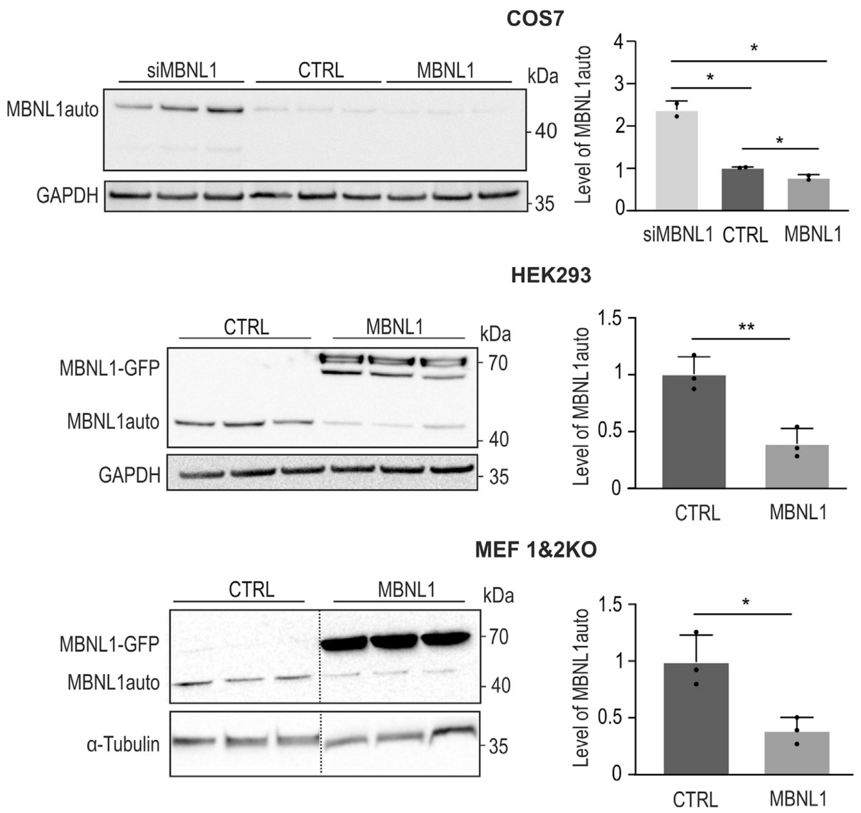

Figure 2. Biosynthesis of the MBNL1auto depends on the available pool of MBNLs

Western blot analysis of MBNL1auto in COS7, HEK-293, and MEF cells with *Mbnl1* and *Mbnl2* knockout. Bars represent average signal from n = 3 independent experiments for each group normalized to GAPDH in COS7 and HEK-293 cells and to α-tubulin in MEF cells. Anti-FLAG antibody staining was carried out as FLAG sequence is fused to the C-terminal end of both constructs. Unpaired Student's t test was used to calculate statistical significance: *$p < 0.05$; **$p < 0.01$.

respectively, and mouse embryonic fibroblasts with full knockout of *Mbnl1* and *Mbnl2* (MEF-1&2KO).[38] These cells were co-transfected with either MBNL1-GFP to increase the pool of MBNLs or GFP used as a negative control (Figure 2). Overexpressed MBNL1-GFP, similar to endogenous MBNLs, can bind to the regulatory element within the intron of MB22#1 pre-mRNA, leading to ex22 inclusion and premature termination of translation (ex22 contains a stop codon), which decreases the level of MBNL1auto. Moreover, COS7 cells were also transfected with small interfering RNA (siRNA) against *MBNL1* (siMBNL1), targeting the sequence within the 3′ UTR to not disturb expression of the MB22 construct. As expected, siMBNL1 treatment significantly increased the level of MBNL1auto by about 2.5 times. On the other hand, the overexpression of MBNL1-GFP has a negative effect on the level of protein from the MB22#1 construct in each tested cell line (Figure 2), leading to an about 3-fold decrease of MBNL1auto in HEK293 and MEF-1&2KO cells. Taken together, these results indicate that production of MBNL1auto strongly depends on the changing level of the MBNL pool in a broad range of MBNL concentrations.

The level of MBNL1auto is homogeneous in cells

To achieve insight into the ability of the MBNL1auto protein to autoregulate, we co-transfected COS7 with different amounts of construct encoding either MBNL1auto or MBNL1-GFP (100–1,000 ng) and minigenes containing MBNL-sensitive alternative exons (*MBNL1* ex5, *Nfix* ex7, *Atp2a1* ex22) (Figures 3A and 3B). This approach gave us the comparison of MBNL1 proteins that came from either autoregulated or non-autoregulated constructs on depth and dynamics of alternative splicing regulation. Results of RT-PCR assay showed that the percentage of inclusion of ex22 from MB22#1 is indeed not sensitive to the amount of transfected construct (Figure 3A). Moreover, increasing amounts of transfected MB22#1 did not significantly differ in the regulation of three other alternative exons (Figure 3B). In contrast, MBNL1-GFP showed concentration-dependent splicing regulation, reaching saturation for the highest amount of overexpression construct (Figure 3B). A different pattern of ex22 inclusion from *Atp2a1* minigene does not demonstrate better activity of MBNL1-GFP than the MBNL1auto, as this minigene responds to low levels of MBNL1 overexpression. Collectively, these results suggest that expression of MBNL1auto can reach a certain maximal level independently from amount of MB22 genetic construct delivered into cells.

To directly measure the diversity of MBNL1auto-GFP production in MB22#2-GFP-transfected COS7 cells, we utilized the flow cytometry approach (Figure S3A). Quantification on resolution of a single cell performed in two independent experiments revealed that the level of MBNL1auto-GFP is more homogeneous (4-times difference in a GFP signal between the 25th and 75th percentiles of analyzed cells) than the level of MBNL1-GFP (13-times difference) which was used as a negative control (Figures 3C and S3B). We confirmed an equal level of MBNL1auto-GFP using confocal microscopy (Figure 3D). Taken together, these findings revealed that MBNL1auto-GFP shows homogeneous expression in transfected cells.

MBNL1auto corrects alternative splicing abnormalities in DM1 cell models

To investigate therapeutic potential of MB22, we first studied if the MBNL1auto-GFP protein can bind to the CUG^{exp} repeats. COS7 cells were co-transfected with a MB22#2 construct expressing MBNL1auto fused with GFP and either a construct expressing a mutant *DMKP* mRNA fragment containing ex11–ex15 with 960 interrupted CUG repeats in the 3′ UTR (CUG_{960}) or a normal *DMPK* fragment without

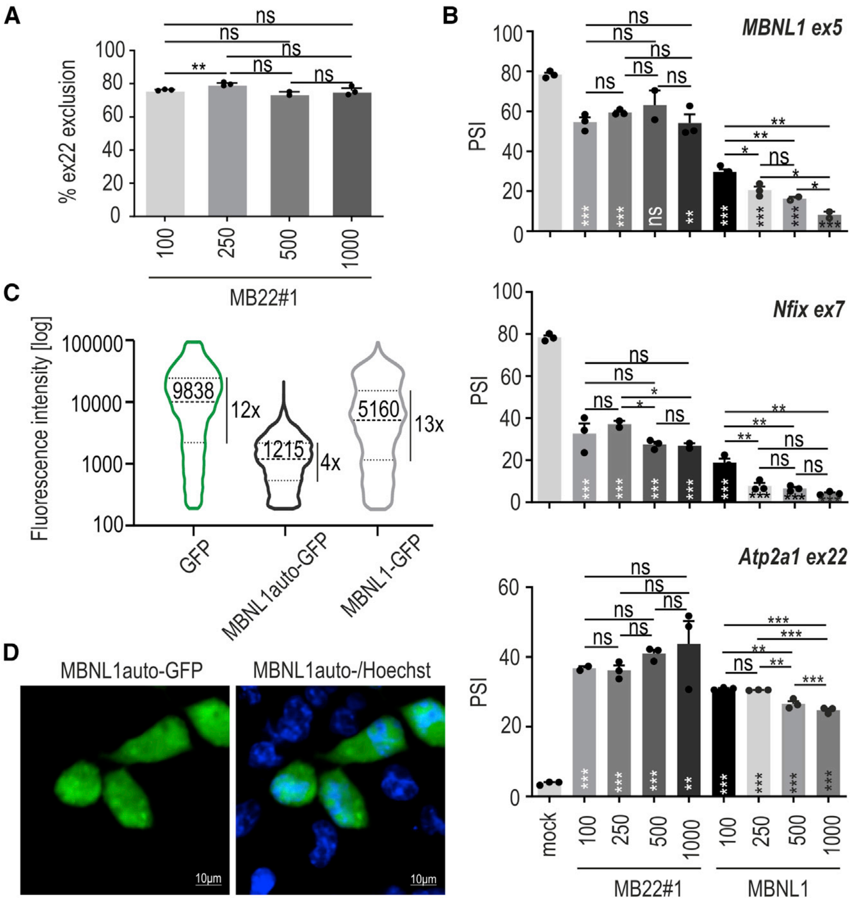

Figure 3. The autoregulatory potential and expression homogeneity of MB22 construct

(A) The percentage of alternative ex22 exclusion from mRNA encoding for MBNL1auto after treatment of COS7 cells with different amounts of MB22#1, ranging from 100 to 1,000 ng/mL culture medium; n = 3. (B) The dose-dependent inclusion of alternative exons of three different MBNL-sensitive minigenes after treatment of COS7 cells with MB22#1- or MBNL1-GFP-overexpression construct (ng/mL); n = 3. (A and B) Unpaired Student's t test; *p < 0.05; **p < 0.01; ***p < 0.001; ns, non-significant. Stars placed on bars denote statistical significance compared with the mock. (C) Violin plots showing the distribution of cells with different fluorescent signals of either GFP, MBNL1auto-GFP, or MBNL1-GFP proteins. COS7 cells were transfected with adequate vectors 48 h prior to flow cytometry analysis. Median fluorescent intensity (black solid line) and 25th and 75th percentiles of signal (dashed lines) are shown. Fold change between 25th and 75th percentiles of signal for all analyses is also indicated. Cells with signal below 200 were rejected from analyses based on results for control experiment for mock-transfected cells. GFP and MBNL1-GFP without autoregulatory cassette state as negative controls. Graphs represent values from n = 4 independent biological replicates for each experimental condition; N = 13,942 (GFP), N = 6,760 (MBNL1auto-GFP), and N = 10,793 (MBNL1-GFP) cells. (D) Representative confocal microscopy image showing localization of MBNL1auto-GFP (green) in COS7 cells transfected with MB22#2-GFP. Nuclei were stained with Hoechst (blue); scale bar, 10 μm.

repeats (CUG_0). In cells with CUG_{960}, the fluorescent signal from MBNL1auto-GFP protein is located mostly in the ribonuclear foci. In contrast, diffuse distribution and, again, homogeneous GFP signals between cells were observed in a control experiment in cells with CUG_0 (Figure 4A). Moreover, we also performed RNA fluorescence *in situ* hybridization (FISH), which also confirmed co-localization of MBNL1auto-GFP with CUG^{exp}, using a probe labeled with Cy-3, which detects CUG^{exp} (Figure S4). These experiments showed that MBNL1auto, by binding to CUG^{exp} in cell nuclei, can replace endogenous MBNLs from sequestration.

Therefore, next we looked at the potential of MBNL1auto on the correction of MBNL-sensitive alternative splicing in two DM1 cell models. In the first, COS7 cells were co-transfected with MB22#1 and mutant or normal *DMPK*-expressing constructs (CUG_{960} or CUG_0) together with two MBNL-sensitive minigenes (*Nfix* ex7, *Atp2a1* ex22). RT-PCR analyses showed that the presence of MBNL1auto leads to the partial, but significant, rescue of all tested DM1-specific alternative splicing events (Figure 4B). To further support these results, we also utilized other cellular models with siRNA-induced insufficiency of MBNLs (si*MBNL1*; as described above). Cells with silenced endogenous *MBNL1* were co-transfected with MB22#1 and the same splicing minigenes (*Nfix* ex7, *Atp2a1* ex22). We confirmed that si*MBNL1* efficiently knocked down *MBNL1* (Figure S5) and significantly increased the level of MBNL1auto (Figure 2). Then, using splicing-specific RT-PCR assays, we demonstrated that MBNL1auto rescued the pathogenic missplicing triggered by MBNL1 deficiency (Figure 4C).

Lentiviral-based production of MBNL1auto corrects splicing abnormalities in cells derived from patients with DM1

The promising results obtained in the two DM models described above encouraged us to assess the therapeutic potential of MB22 in two different cells derived from patients with DM1 that expressed *DMPK* transcripts with very long CUG^{exp} (DM1-1 and DM1-2). Previously, we showed that these cells manifest DM1-specific molecular phenotypes like the formation of CUG^{exp} nuclear foci, sequestration of MBNLs, and missplicing of several transcripts.[25,39] In this study, we utilized lentiviral vectors encoding MBNL1auto-GFP from autoregulatory MB22#2-GFP or GFP used as a negative control. The fusion with GFP was used to monitor the efficacy of transduction. The fibroblast from a healthy individual was used as a control cell line (non-DM). Twelve days after transduction, we checked the expression level of the *DMPK* gene to assess the potential effect of the production of

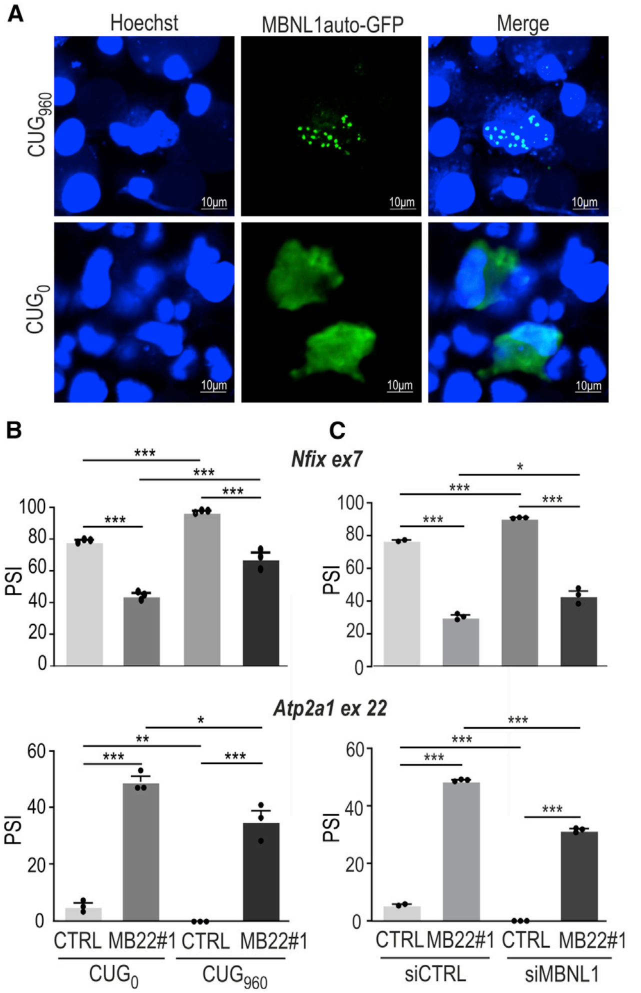

Figure 4. The therapeutic potential of MB22 autoregulated construct in DM models

(A) Representative confocal images showing the nuclear foci containing MBNL1auto-GFP in cells expressing a mutant *DMPK* fragment with CUG_{960} (top panel). In cells expressing a normal DMPK fragment without CUG repeats, MBNL1auto-GFP is distributed equally (bottom panel); scale bar, 10 μm. (B) Results of RT-PCR analyses showing changes in the regulation of two MBNL-dependent exons from *Nfix* ex7 and *Atp2a1* ex22 minigenes. Splicing changes are expressed as the percent spliced in (PSI). Cells were co-transfected with MB22#1 or control GFP construct (CTRL) and either mutant (CUG_{960}) or normal (CUG_0) *DMPK*-fragment-expressing constructs. (C) As in (B) but for cells treated with either control siRNA (siCTRL) or siRNA targeting the 3′ UTR of *MBNL1* (siMBNL1). These cells were then transfected with either GFP or MB22#1 construct. The percentage of mRNA isoform with inclusion of alternative exon was calculated using the inverse of PSI parameter, which demonstrates the portion of mRNA with an included alternative exon. Bars represent average PSI from three independent experiments (with exception of n = 2 for siCTRL + GFP condition) with SD. (B and C) Unpaired Student's t test was used to calculate statistical significance. *$p < 0.05$; **$p < 0.01$; ***$p < 0.001$; ns, non-significant.

MBNL1auto. The quantitative real-time RT-PCR analysis showed no differences in the steady-state level of *DMPK* mRNA in samples treated with different vectors (Figure 5A). Next, we evaluated alternative splicing changes of eight mRNAs that are known to be MBNL sensitive: *INSR* ex11, *FLNB* ex31, *MYO5A* ex33, *MBNL2* ex5, *MBNL2* ex7, *MBNL1* ex1, *NCOR2* ex19, and *PHKA1* ex19. All of them showed significant correction in both DM1 cell lines (Figures 5B and S6). Importantly, no significant splicing changes were observed in the non-DM1 cell line treated with MB22#2-GFP lentivirus (the only exception is a small change of ex19 inclusion in *PHKA1*). Cumulatively, all these data strongly suggest that the MBNL1auto protein, whose production is autoregulated on the level of alternative splicing of its mRNA, has the ability to rescue alternative splicing alterations in different DM1 models.

DISCUSSION

Gene therapy is promising strategy for many incurable diseases. It involves the delivery of new genetic material to patient cells to prevent or slow down development of a particular disease. This kind of treatment enables the efficient cure for some monogenic disorders, including spinal muscular atrophy (Zolgensma).[40] Overexpression can rescue proper protein function but, when uncontrolled, may also trigger undesirable effects.[41,42] Therefore, an important limitation to overcome in the gene therapy approach is to adjust the level of overexpressed protein to prevent toxicity but enable the therapeutic effect.

During different stages of tissue differentiation and development, the level of the MBNL pool is crucial for proper alternative splicing regulation. Expression of *MBNL1* and *MBNL2* increases during both embryonic and postnatal stages of development, with the highest level of MBNL1 in adult muscles and the highest MBNL2 in the adult brain.[43,44] All three MBNL paralogs regulate the same splicing events; however, among them, fast and slow responders exist. Some alternative exons respond to low and some to high levels of MBNLs, and the depth of exon inclusion/exclusion could be regulated in a high range of concentration of these proteins.[45,46] Therefore, fine-tuning of the MBNL pool is important for proper function of different tissues.

In DM, the activity of all MBNL paralogs is significantly lower due to sequestration of these proteins on toxic CUG^{exp} or $CCUG^{exp}$,[14,15] and the increase of MBNLs is considered a potential therapeutic strategy, including the application of gene therapy tools.[47] It was shown that AAV-based production of the MBNL1 protein or its truncated variant rescued muscle hyperexcitability and splicing defects of many MBNL-sensitive genes.[30,31] Moreover, expression of *MBNL1* from a transgene in knockin mouse models also improved the DM-specific phenotypes.[32] On the other hand, overexpression of full-length MBNL1 induced by intramuscular injection of AAV vector in WT mice showed significant muscle damage.[31] Also, transgenic mice overexpressing MBNL1 from a transgene demonstrated significantly decreased body weight, grip strength, run distance, and heart failure compared with WT littermates[33] or even reduced survival.[32] Moreover, uncontrolled and unbalanced overexpression might lead to the excessive activity of MBNLs in some treated muscle fibers or other tissues and, consequently, change the metabolism of many RNA.

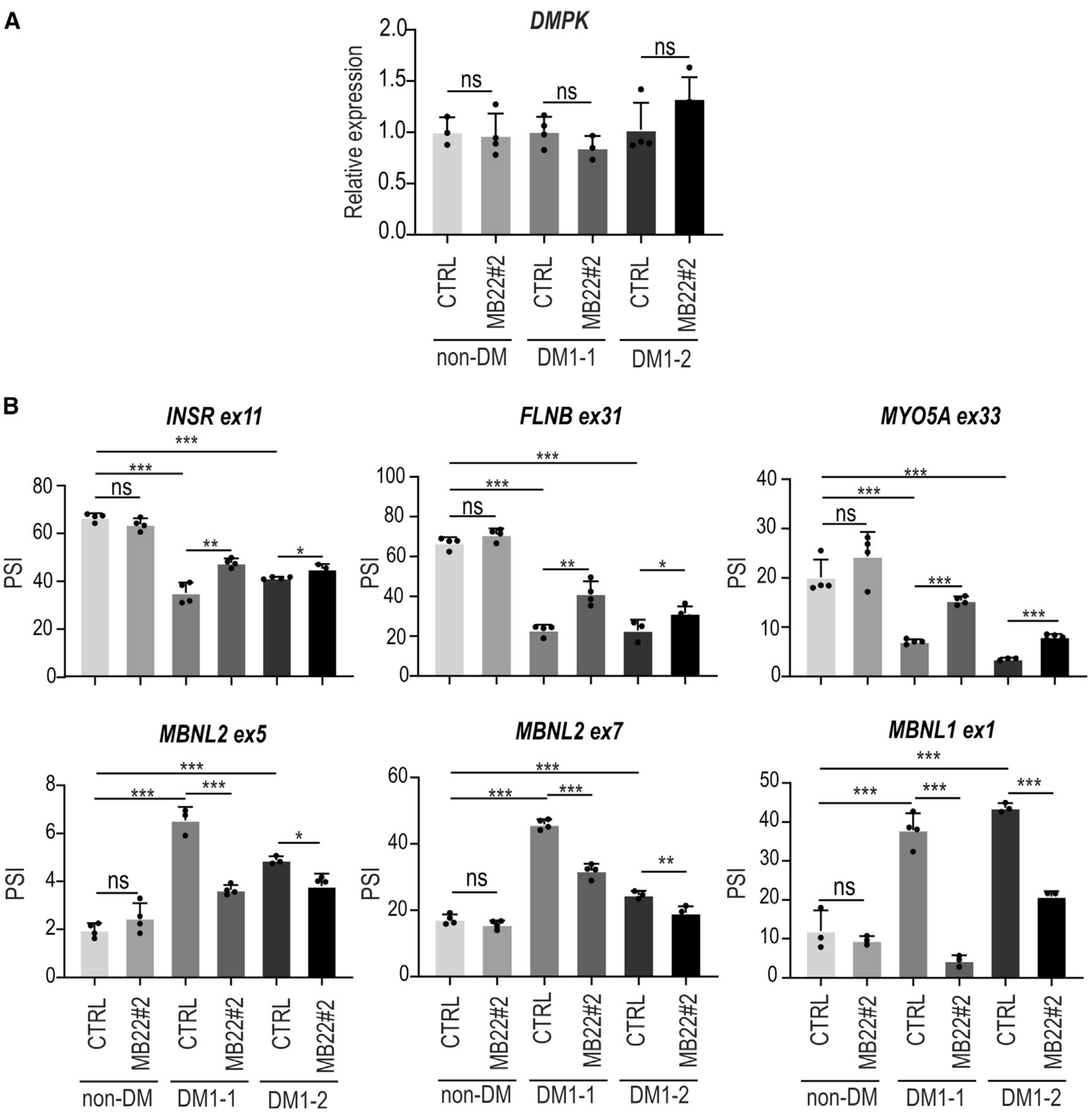

Figure 5. Correction of pathogenic missplicing in DM1 cells treated with MB22 lentiviruses

(A) Results of quantitative real-time RT-PCR analysis showing relative expression of *DMPK* (normalized to *GAPDH*) in three different cell lines: fibroblasts derived from healthy individual (non-DM) and two patients with DM1 (DM1-1 and DM1-2) treated with either control (CTRL) lentiviral vector or lentivirus containing the MB22#2-GFP sequence 12 days from cell transduction. (B) Results of RT-PCR-based analyses of alternative splicing changes in cells described in (A). Changes in the inclusion of positively (top panel) and negatively (bottom panel) regulated MBNL-dependent alternative exons are shown for six transcripts affected in DM1. Splicing changes are expressed as PSI. Bars represent average from 3 to 4 independent experiments (dots); unpaired Student's t test; *p < 0.05; **p < 0.01; ***p < 0.001; ns, non-significant.

Therefore, in this study we decided to develop the self-regulating MB22 overexpression construct for fine-tuning MBNL1auto production, which depends on the endogenous MBNL pool available in the cell. In designing MBNL1auto, we selected the sequence of the 41 kDa isoform of MBNL1. This isoform is deprived of alternative ex5, which is responsible for nuclear localization of the protein, but contains alternative ex7, which increases splicing activity of the protein.[4,48,49] Importantly, this isoform is localized in both the nucleus and cytoplasm and can regulate not only alternative splicing but also other RNA metabolism stages[3] and is one of major isoforms present in skeletal and cardiac muscles in adults.[46] It was already demonstrated that the MBNL1 isoform with ex7 can efficiently bind to CUG^{exp} and $CCUG^{exp}$.[46]

Previous studies of ours and others showed that activity of MBNLs can be autoregulated on the level of alternative splicing.[4,50] Therefore, we decided to design the autoregulatory MB22 construct, whose expression is sensitive to the MBNL pool, by adding the intron/ex22/intron sequence from *ATP2A1* between the sequence encoding MBNL1 (Figure 1A). MBNL-dependent alternative splicing of pre-mRNA from this

construct is regulated by the efficiency of the inclusion of alternative ex22 containing an in-frame stop codon, and the inclusion of this exon leads to the production of a non-functional short protein.

As proposed in this study, an autoregulated MB22 construct enables the restricted expression of MBNL1auto (Figure 2), which is sufficient for the rescue of DM-specific alternative splicing defects in cellular models with insufficiency of MBNLs induced by either silencing of *MBNL1* or overexpression of toxic CUG^{exp} (Figure 4).

In muscles of patients with DM1 and DM2, the size of CTG or CCTG repeat expansion differs significantly between patients but also between different parts of the same muscle of the same individual, due to somatic instability of repeat tract.[12,18,21,22] Hence, the sequestration of MBNL proteins is highly heterogeneous. Therefore, we hypothesized that unequal sequestration of the MBNL pool can be buffered by self-regulated overexpression of MBNL1auto. This was partially demonstrated in experiments with transfection-based and uncontrolled delivery of the MB22-GFP construct to model cells (Figure 3). Using conventional overexpression systems, we can expect high heterogeneity in the number of transcripts and proteins generated from the transgene, e.g., as a result of different numbers of copies of plasmid per single cell. Quantitative flow cytometry experiments and microscopic analyses (Figures 3C, S3B, and S3D), as well as monitoring of MBNL-sensitive splicing patterns (Figure 3B), showed that the production of MBNL1auto is homogeneous from cell to cell even after imbalanced delivery of the MB22 genetic construct. Importantly, the buffering level of MBNL1auto leads to significant correction of splicing abnormalities in cellular models of DM1 (Figures 4 and 5).

The therapeutic effect would be caused partially by binding of MBNL1auto to CUG^{exp} and replacement of the endogenous pool of MBNLs from sequestration (Figures 4A and S4) and partially by the splicing activity of MBNL1auto itself. We can predict that the required level of MBNL1auto in skeletal muscles of DM would be different than in the model cells used in this study. Therefore, in further studies, the MBNL-sensitive RNA regulatory element in MB22 can be easily replaced, giving the opportunity to better adjust the buffering level of the MBNL1auto. The regulatory element with lower affinity to MBNL proteins can be used to reduce sensitivity of ex22 inclusion and, consequently, increase the levels of arranged protein, keeping still the control on excess activity of MBNLs. Moreover, the use of a well-selected tissue-specific promoter and a proper delivery system could be adjusted.

Taken together, our results highlight the utility of autoregulated overexpression of MBNL1auto as a potential therapeutic tool in DM1, DM2, and other diseases in which MBNL proteins are sequestrated or insufficient.

MATERIALS AND METHODS

Genetic constructs

The MB22 construct was prepared by PCR amplification of an *ATP2A1* gene fragment from human genomic DNA (for primers, see Table S1) and cloning into the previously described pEGFP-C1-MBNL1-41 vector for MBNL1 overexpression[46] with removed GFP sequence. The *MBNL1* sequence contains alternative ex7 and is deprived of alternative ex5, which contains a nuclear localization signal (Figure S1). The MB22-del and MB22#2 constructs were obtained by deletion of the fragment with MBNL-binding motifs and the replacement of the WT fragment with the 4xUGCU sequence, respectively. The MB22#2-GFP was prepared by an amplified EGFP sequence from pEGFP-C1 (CloneTech) and fusion to the C-terminal end of *MBNL1*. All the above constructs were prepared using the In-Fusion Cloning method. The desired fragments were amplified using CloneAmp HiFi PCR Premix (Takarabio) and cloned using NEBuilder HiFi DNA Assembly Master Mix (New England Biolabs) following the manufacturer's instructions. The pEGFP-C1-MBNL1-41 vector used in this study (referred to as MBNL1-GFP overexpression vector) was previously described.[50] The DT960 (CUG_{960}) and DT0 (CUG_0) vectors were a gift (Prof. Thomas Cooper, Baylor College of Medicine) and were already described.[25,51] The pEGFP-C1 vector (CloneTech) was used as a control and is referred to as GFP. The *Atp2a1* ex22[52] and *Nfix* ex7 minigenes, gifts from Prof. Manuel Ares, University of California, Santa Cruz, were described earlier.[8,52] The *MBNL1* ex5 minigene is described in the manuscript (Taylor K. et al., unpublished data). The sequences of primers used in cloning are listed in Table S1. The lentiviral vectors were prepared by an external company (Viral Core Facility) by cloning the sequence of the MB22#2-GFP construct under the control of a cytomegalovirus (CMV) promoter. The lentiviral vector (BL-0533) containing a GFP sequence under the same promoter was used as a control.

Cell culture, transfection, and transduction

The monkey COS7, human HEK293, and mouse embryonic fibroblast (MEF) cells were grown in a high-glucose DMEM medium with L-glutamine (Biowest) supplemented with 10% fetal bovine serum (Thermo Fisher Scientific) and 1% antibiotic/antimycotic (Life Technologies) at 37°C in 5% CO_2. Fibroblasts derived from patients with DM1 (cell lines GM04033 and GM03989 expressing *DMPK* transcripts with ~1,000 and ~2,000 CUG repeats, respectively) and control fibroblasts derived from non-DM1 patient (cell line GM07492) were purchased from the Coriell Cell Repositories. Fibroblasts were grown in Eagle's minimal essential medium (EMEM) (Biowest) supplemented with 10% fetal bovine serum (FBS) (Thermo Fisher Scientific), 1% antibiotic antimycotic (Life Technologies), and 1% non-essential amino acids solution (Sigma) in 5% CO_2 at 37°C. MEF 1&2KO cells were gifts from Maurice Swanson at the University of Florida. For transfection, cells were plated in 12-well plates and transfected at ~80% confluency using Lipofectamine 3000 (Thermo Fisher Scientific) according to the manufacturer's instructions. Single transfection with siRNA against the 3′ UTR fragment of the *MBNL1* sequence or control siRNA (synthesized by Sigma-Aldrich) was performed using Lipofectamine RNAiMAX (Thermo Fisher Scientific) at 50 nM concentration (sequence of siRNA duplexes are specified in Table S2). After 24 h, cells were transfected with plasmids. Co-transfection was conducted in a 1:1 ratio with MB22 construct and pEGFP-C1 or pEGFP-C1-MBNL1-41 vector; the same ratio was used in the

experiments with DT960 vector. The co-transfection with minigenes was made in a 1:10 ratio (minigene: construct). For all experiments, the total amount of DNA added to the cells was 1 μg/mL of cell culture medium, except for experiments with different plasmid concentrations, which are specified in figure legend (Figure 3B). The cells were harvested 48 h after transfection or 72 h after the experiment with siRNA treatment. The samples referred to as a mock are lipofectamine-treated cells. For transduction, cells were plated in 48-well plates. Lentiviral vectors with a concentration of 10^7 particles/mL at a multiplicity of infection (MOI) 1 for control and MOI 10 for MB22#2-GFP in an appropriate amount of growth medium were delivered into the cells. After 24 h, the medium was replaced. The cells were harvested after 12 days.

Alternative splicing and real-time qPCR analysis

The cells were harvested using TRIzol Reagent (Thermo Fisher Scientific), and total RNA was isolated using Total RNA Zol-Out D (A&A Biotechnology) according to the manufacturer's protocol. cDNA was synthesized using TranScriba Kit (A&A Biotechnology) with Random Primers (A&A Biotechnology) according to the manufacturer's protocol. PCR was performed using GoTaq DNA Polymerase (Promega), and primers are listed in Table S3. PCR products were separated in 1%–2% agarose gel with ethidium bromide. The images were captured using G:Box EF2 (Syngene) and analyzed using GeneTools (Syngene) (Figures S7A, S7B, S9A, S9B, S10, and S12). Percent sliced in (PSI) was calculated based on signals of two bands, corresponding to the PCR product containing or missing alternative exons, according to the following formula (isoform with included exon*100)/(isoforms with included exon + excluded exon). Quantitative real-time RT-PCRs were performed in a QuantStudio 7 Flex System (Thermo Fisher Scientific) using Maxima SYBR Green/ROX qPCR Master Mix (Thermo Fisher Scientific) according to the manufactures' instructions. Targets were amplified with primers listed in Table S3 at 58°C–60°C annealing temperature. Ct values were normalized against *GAPDH*. Fold differences in expression level were calculated according to the $2-\Delta\Delta$Ct method.

Western blot

Cells were lysed with radioimmunoprecipitation assay (RIPA) buffer (150 mM NaCl, 50 mM Tris-HCl [pH 8.0], 1 mM ethylenediaminetetraacetic acid [EDTA], 0.5% NP-40, 0.5% Triton X-100, 0.5% sodium deoxycholate, 0.1% sodium dodecyl sulfate [SDS]) supplemented with Halt Protease Inhibitor Cocktail (Thermo Fisher Scientific). Lysates were incubated on ice and vortexed followed by centrifugation at 15,000 × *g* at 4°C for 15 min. Concentration of protein in cell extracts were measured by Pierce BCA Protein Assay Kit (Thermo Fisher Scientific). Samples were heated with standard sample buffer at 95°C for 5 min. Electrophoresis and wet transfer were performed with the use of the Mini-PROTEAN Tetra System (Bio-Rad). Protein extracts (25–40 μg) were separated on 10% SDS polyacrylamide gels in Laemmli buffer and transferred to nitrocellulose membrane (Sigma-Aldrich) (1 h, 100 V) in Laemmli buffer with 20% methanol. Membranes were blocked for 1 h in 5% Skim Milk Powder (Sigma-Aldrich) in TBST buffer (Tris-buffered saline [TBS], 0.1% Tween 20). Membranes were incubated with a primary antibody against FLAG (A8592, Sigma-Aldrich) 1:1,000, GAPDH (sc-47724, Santa Cruz) 1:10,000, or mCherry (5F8, ChromoTek) 1:1,000 in 5% Skim Milk Powder in TBST for 1 h at room temperature. Membranes were washed in TBST and incubated with secondary antibodies conjugated with horseradish peroxidase, anti-mouse (A9044, Sigma-Aldrich) 1:20,000, or anti-rat (ab6836, abcam) 1:8,000 in TBST for 1 h at room temperature. Membranes were again washed in TBST and detected using Immobilon Forte Western HRP substrate (Sigma-Aldrich). Images were captured using G:Box Chemi-XR5 (Syngene) (Figures S7B, S8, and S11) and quantified using Multi Gauge 3.0 software (Fujifilm).

Microscopic analysis

The confocal microscopy was used to analyze sequestration of MBNL1auto-GFP to the CUG^{exp} ribonuclear foci and to monitor homogeneity of the level of this protein in cells. COS7 cells were plated in μ-Slide 8 well (ibidi) after 48 h posttransfection with MB22#2-GFP, and (CUG_{960}) or (CUG_0) medium was replaced with PBS with diluted Hoechst (Thermo Fisher Scientific), 1:2,000, and incubated for 5 min, protected from light. Images were captured with the Nikon A1Rsi confocal microscope with Nikon Apo ×40 WI λ S DIC N2 objective. GFP, Hoechst, and Cy3 were excited with 488 nm Argon-Ion and 405 and 561 nm diode lasers, respectively. For detection, dichroic mirrors of 405/488/561 nm with spectral filters of 525/50, 595/50, and 450/50 nm were used.

Flow cytometry

For flow cytometry experiments, cells were analyzed 48 h posttransfection with either GFP, MB22#2-GFP, or MBNL1-GFP plasmid. Culture medium was removed, and cells were washed with PBS, trypsinized, collected, and centrifuged for 5 min at 300 × *g*. Cell pellet was suspended in 400 μL PBS. The 100 μL cell suspension was diluted with 100 μL PBS and analyzed with guava easyCyteTM HT flow cytometer and guavaSoft software (Luminex). GFP fluorescence was excited by a 488 nm laser and detected at 525/30 nm. The threshold for GFP-positive cells was set based on the signal from mock-transfected cells. For each sample, 5,000 events were collected. The single event was referred to as single cell. The gating strategy is presented in Figure S3A.

FISH

RNA FISH and immunofluorescence (IF) cells were fixed in 2% PFA/PBS at room temperature for 10 min and washed three times in PBS. Pre-hybridization was performed in 30% formamide and 2× SSC for 10 min, followed by hybridization in buffer containing 30% formamide, 2× SSC, 0.02% BSA, 66 μg/mL yeast tRNA, 10% dextran sulfate, 2 mM vanadyl ribonucleoside complex, and 2 ng/μL DNA/LNA probe $(CAG)_6$-CA. The probe was labeled at the 5′ end with Cy3 and modified at positions 2, 5, 8, 13, 16, and 19 with LNA. Posthybridization washing was done in 30% formamide and 2× SSC at 45°C for 30 min followed by 1× SSC at 37°C for the next 30 min. Slides were mounted in Vectashield medium (Vector Laboratories, Burlingame, CA, USA) with DAPI.

Statistical analysis

Group data are expressed as the means ± standard deviation (SD). The statistical significance was determined by unpaired, two-tailed Student's t test using Prism software v.8 (GraphPad): *$p < 0.05$; **$p < 0.01$; ***$p < 0.001$; ns, non-significant. All analyses are based on at least three independent biological replicates (exceptions are indicated in the figure legends), and whole experiments were repeated at least twice to confirm obtained results.

DATA AVAILABILITY

This study did not generate/analyze datasets or code.

SUPPLEMENTAL INFORMATION

Supplemental information can be found online at https://doi.org/10.1016/j.omtn.2022.10.023.

ACKNOWLEDGMENTS

We thank Dr. Katarzyna Taylor for sharing the *MBNL1* ex5 minigene and Dr. Wojciech Kwiatkowski for assistance with confocal microscopy. This work was supported by the Foundation for Polish Science TEAM program co-financed by the European Union within the European Regional Development Fund (POIR.04.04.00-00-5C0C/17-00 to K.S.), Polish National Science Center (2020/38/A/NZ3/00498 to K.S.), the Ministry of Science and Higher Education of the Republic of Poland, from the quality-promoting subsidy, under the Leading National Research Center (KNOW) program for the years 2012–2017 (KNOW RNA Research Center in Poznan; 01/KNOW2/2014). Funding for open access charge: Initiative of Excellence–Research University (05/IDUB/2019/94) at Adam Mickiewicz University, Poznan, Poland.

AUTHOR CONTRIBUTIONS

Conception and design of the experiments, K.S.; performed the experiments, Z.R.; analysis of data, K.S. and Z.R.; writing and revising the manuscript and figures, K.S. and Z.R.

DECLARATION OF INTERESTS

The authors declare no conflicts of interest.

REFERENCES

1. Ho, T.H., Charlet-B, N., Poulos, M.G., Singh, G., Swanson, M.S., and Cooper, T.A. (2004). Muscleblind proteins regulate alternative splicing. EMBO J. *23*, 3103–3112. https://doi.org/10.1038/sj.emboj.7600300.

2. Batra, R., Manchanda, M., and Swanson, M.S. (2015). Global insights into alternative polyadenylation regulation. RNA Biol. *12*, 597–602. https://doi.org/10.1080/15476286.2015.1040974.

3. Wang, E.T., Cody, N.A.L., Jog, S., Biancolella, M., Wang, T.T., Treacy, D.J., Luo, S., Schroth, G.P., Housman, D.E., Reddy, S., et al. (2012). Transcriptome-wide regulation of pre-mRNA splicing and mRNA localization by muscleblind proteins. Cell *150*, 710–724. https://doi.org/10.1016/j.cell.2012.06.041.

4. Fernandez-Costa, J.M., Llamusi, M.B., Garcia-Lopez, A., and Artero, R. (2011). Alternative splicing regulation by Muscleblind proteins: from development to disease. Biol. Rev. Camb. Philos. Soc. *86*, 947–958. https://doi.org/10.1111/j.1469-185X.2011.00180.x.

5. Kanadia, R.N., Urbinati, C.R., Crusselle, V.J., Luo, D., Lee, Y.J., Harrison, J.K., Oh, S.P., and Swanson, M.S. (2003). Developmental expression of mouse muscleblind genes Mbnl1, Mbnl2 and Mbnl3. Gene Expr. Patterns *3*, 459–462. https://doi.org/10.1016/S1567-133X(03)00064-4.

6. Fardaei, M., Rogers, M.T., Thorpe, H.M., Larkin, K., Hamshere, M.G., Harper, P.S., and Brook, J.D. (2002). Three proteins, MBNL, MBLL and MBXL, co-localize in vivo with nuclear foci of expanded-repeat transcripts in DM1 and DM2 cells. Hum. Mol. Genet. *11*, 805–814. https://doi.org/10.1093/hmg/11.7.805.

7. Teplova, M., and Patel, D.J. (2008). Structural insights into RNA recognition by the alternative-splicing regulator muscleblind-like MBNL1. Nat. Struct. Mol. Biol. *15*, 1343–1351. https://doi.org/10.1038/nsmb.1519.

8. Du, H., Cline, M.S., Osborne, R.J., Tuttle, D.L., Clark, T.A., Donohue, J.P., Hall, M.P., Shiue, L., Swanson, M.S., Thornton, C.A., and Ares, M., Jr. (2010). Aberrant alternative splicing and extracellular matrix gene expression in mouse models of myotonic dystrophy. Nat. Struct. Mol. Biol. *17*, 187–193. https://doi.org/10.1038/nsmb.1720.

9. Goers, E.S., Purcell, J., Voelker, R.B., Gates, D.P., and Berglund, J.A. (2010). MBNL1 binds GC motifs embedded in pyrimidines to regulate alternative splicing. Nucleic Acids Res. *38*, 2467–2484. https://doi.org/10.1093/nar/gkp1209.

10. Harper, P.S. (2001). Myotonic dystrophy, 3rd ed. (WB Saunders).

11. Brook, J.D., McCurrach, M.E., Harley, H.G., Buckler, A.J., Church, D., Aburatani, H., Hunter, K., Stanton, V.P., Thirion, J.-P., Hudson, T., et al. (1992). Molecular basis of myotonic dystrophy: expansion of a trinucleotide (CTG) repeat at the 3′ end of a transcript encoding a protein kinase family member. Cell *68*, 799–808. https://doi.org/10.1016/0092-8674(92)90154-5.

12. Liquori, C.L., Ricker, K., Moseley, M.L., Jacobsen, J.F., Kress, W., Naylor, S.L., Day, J.W., and Ranum, L.P. (2001). Myotonic dystrophy type 2 caused by a CCTG expansion in intron 1 of *ZNF9*. Science *293*, 864–867. https://doi.org/10.1126/science.1062125.

13. Miller, J.W., Urbinati, C.R., Teng-Umnuay, P., Stenberg, M.G., Byrne, B.J., Thornton, C.A., and Swanson, M.S. (2000). Recruitment of human muscleblind proteins to (CUG)n expansions associated with myotonic dystrophy. EMBO J. *19*, 4439–4448. https://doi.org/10.1093/emboj/19.17.4439.

14. Sobczak, K., de Mezer, M., Michlewski, G., Krol, J., and Krzyzosiak, W.J. (2003). RNA structure of trinucleotide repeats associated with human neurological diseases. Nucleic Acids Res. *31*, 5469–5482. https://doi.org/10.1093/nar/gkg766.

15. Fardaei, M., Larkin, K., Brook, J.D., and Hamshere, M.G. (2001). In vivo co-localisation of MBNL protein with DMPK expanded-repeat transcripts. Nucleic Acids Res. *29*, 2766–2771. https://doi.org/10.1093/nar/29.13.2766.

16. Jiang, H., Mankodi, A., Swanson, M.S., Moxley, R.T., and Thornton, C.A. (2004). Myotonic dystrophy type 1 is associated with nuclear foci of mutant RNA, sequestration of muscleblind proteins and deregulated alternative splicing in neurons. Hum. Mol. Genet. *13*, 3079–3088. https://doi.org/10.1093/hmg/ddh327.

17. Martorell, L., Monckton, D.G., Gamez, J., Johnson, K.J., Gich, I., Lopez De Munain, A., and Baiget, M. (1998). Progression of somatic CTG repeat length heterogeneity in the blood cells of myotonic dystrophy patients. Hum. Mol. Genet. *7*, 307–312. https://doi.org/10.1093/hmg/7.2.307.

18. Wong, L.J., Ashizawa, T., Monckton, D.G., Caskey, C.T., and Richards, C.S. (1995). Somatic heterogeneity of the CTG repeat in myotonic dystrophy is age and size dependent. Am. J. Hum. Genet. *56*, 114–122.

19. Thornton, C.A., Johnson, K., and Moxley, R.T. (1994). Myotonic dystrophy patients have larger CTG expansions in skeletal muscle than in leukocytes. Ann. Neurol. *35*, 104–107. https://doi.org/10.1002/ana.410350116.

20. Zatz, M., Passos-Bueno, M.R., Cerqueira, A., Marie, S.K., Vainzof, M., and Pavanello, R.C. (1995). Analysis of the CTG repeat in skeletal muscle of young and adult myotonic dystrophy patients: when does the expansion occur? Hum. Mol. Genet. *4*, 401–406. https://doi.org/10.1093/hmg/4.3.401.

21. Monckton, D.G., Wong, L.J., Ashizawa, T., and Caskey, C.T. (1995). Somatic mosaicism, germline expansions, germline reversions and intergenerational reductions in myotonic dystrophy males: small pool PCR analyses. Hum. Mol. Genet. *4*, 1–8. https://doi.org/10.1093/hmg/4.1.1.

22. Ballester-Lopez, A., Núñez-Manchón, J., Koehorst, E., Linares-Pardo, I., Almendrote, M., Lucente, G., Guanyabens, N., Lopez-Osias, M., Suárez-Mesa, A., Hanick, S.A., et al. (2020). Three-dimensional imaging in myotonic dystrophy type 1. Neurol. Genet. *6*, 4844. https://doi.org/10.1212/NXG.0000000000000484.

23. Jenquin, J.R., O'Brien, A.P., Poukalov, K., Lu, Y., Frias, J.A., Shorrock, H.K., Richardson, J.I., Mazdiyasni, H., Yang, H., Huigens, R.W., et al. (2022). Molecular characterization of myotonic dystrophy fibroblast cell lines for use in small molecule screening. iScience *25*, 104198. https://doi.org/10.1016/j.isci.2022.104198.

24. Alfano, M., de Antoni, L., Centofanti, F., Visconti, V.V., Maestri, S., Degli Esposti, C., Massa, R., D'Apice, M.R., Novelli, G., Delledonne, M., et al. (2022). Characterization of full-length CNBP expanded alleles in myotonic dystrophy type 2 patients by Cas9-mediated enrichment and nanopore sequencing. Elife *11*, e80229. https://doi.org/10.7554/eLife.80229.

25. Wojtkowiak-Szlachcic, A., Taylor, K., Stepniak-Konieczna, E., Sznajder, L.J., Mykowska, A., Sroka, J., Thornton, C.A., and Sobczak, K. (2015). Short antisense-locked nucleic acids (all-LNAs) correct alternative splicing abnormalities in myotonic dystrophy. Nucleic Acids Res. *43*, 3318–3331. https://doi.org/10.1093/nar/gkv163.

26. Nakamori, M., Taylor, K., Mochizuki, H., Sobczak, K., and Takahashi, M.P. (2016). Oral administration of erythromycin decreases RNA toxicity in myotonic dystrophy. Ann. Clin. Transl. Neurol. *3*, 42–54. https://doi.org/10.1002/acn3.271.

27. Christou, M., Wengel, J., Sokratous, K., Kyriacou, K., Nikolaou, G., Phylactou, L.A., and Mastroyiannopoulos, N.P. (2020). Systemic evaluation of chimeric LNA/2′-O-methyl steric blockers for myotonic dystrophy type 1 therapy. Nucleic Acid Ther. *30*, 80–93. https://doi.org/10.1089/nat.2019.0811.

28. Wheeler, T.M., Sobczak, K., Lueck, J.D., Osborne, R.J., Lin, X., Dirksen, R.T., and Thornton, C.A. (2009). Reversal of RNA dominance by displacement of protein sequestered on triplet repeat RNA. Science *325*, 336–339. https://doi.org/10.1126/science.1173110.

29. Chen, G., Masuda, A., Konishi, H., Ohkawara, B., Ito, M., Kinoshita, M., Kiyama, H., Matsuura, T., and Ohno, K. (2016). Phenylbutazone induces expression of MBNL1 and suppresses formation of MBNL1-CUG RNA foci in a mouse model of myotonic dystrophy. Sci. Rep. *6*, 25317–25411. https://doi.org/10.1038/srep25317.

30. Kanadia, R.N., Shin, J., Yuan, Y., Beattie, S.G., Wheeler, T.M., Thornton, C.A., and Swanson, M.S. (2006). Reversal of RNA missplicing and myotonia after muscleblind overexpression in a mouse poly(CUG) model for myotonic dystrophy. Proc. Natl. Acad. Sci. USA *103*, 11748–11753. https://doi.org/10.1073/pnas.0604970103.

31. Arandel, L., Matloka, M., Klein, A.F., Rau, F., Sureau, A., Ney, M., Cordier, A., Kondili, M., Polay-Espinoza, M., Naouar, N., et al. (2022). Reversal of RNA toxicity in myotonic dystrophy via a decoy RNA-binding protein with high affinity for expanded CUG repeats. Nat. Biomed. Eng. *6*, 207–220. https://doi.org/10.1038/s41551-021-00838-2.

32. Chamberlain, C.M., and Ranum, L.P.W. (2012). Mouse model of muscleblind-like 1 overexpression: skeletal muscle effects and therapeutic promise. Hum. Mol. Genet. *21*, 4645–4654. https://doi.org/10.1093/hmg/dds306.

33. Yadava, R.S., Kim, Y.K., Mandal, M., Mahadevan, K., Gladman, J.T., Yu, Q., and Mahadevan, M.S. (2019). MBNL1 overexpression is not sufficient to rescue the phenotypes in a mouse model of RNA toxicity. Hum. Mol. Genet. *28*, 2330–2338. https://doi.org/10.1093/hmg/ddz065.

34. Meola, G. (2013). Clinical aspects, molecular pathomechanisms and management of myotonic dystrophies. Acta Myol. *32*, 154–165.

35. Nakamori, M., Sobczak, K., Puwanant, A., Welle, S., Eichinger, K., Pandya, S., Dekdebrun, J., Heatwole, C.R., McDermott, M.P., Chen, T., et al. (2013). Splicing biomarkers of disease severity in myotonic dystrophy. Ann. Neurol. *74*, 862–872. https://doi.org/10.1002/ana.23992.

36. Hino, S.I., Kondo, S., Sekiya, H., Saito, A., Kanemoto, S., Murakami, T., Chihara, K., Aoki, Y., Nakamori, M., Takahashi, M.P., and Imaizumi, K. (2007). Molecular mechanisms responsible for aberrant splicing of SERCA1 in myotonic dystrophy type 1. Hum. Mol. Genet. *16*, 2834–2843. https://doi.org/10.1093/hmg/ddm239.

37. Taylor, K., Sznajder, L.J., Cywoniuk, P., Thomas, J.D., Swanson, M.S., and Sobczak, K. (2018). MBNL splicing activity depends on RNA binding site structural context. Nucleic Acids Res. *46*, 9119–9133. https://doi.org/10.1093/nar/gky565.

38. Batra, R., Charizanis, K., Manchanda, M., Mohan, A., Li, M., Finn, D.J., Goodwin, M., Zhang, C., Sobczak, K., Thornton, C.A., and Swanson, M.S. (2014). Loss of MBNL leads to disruption of developmentally regulated alternative polyadenylation in RNA-mediated disease. Mol. Cell *56*, 311–322. https://doi.org/10.1016/j.molcel.2014.08.027.

39. Stepniak-Konieczna, E., Konieczny, P., Cywoniuk, P., Dluzewska, J., and Sobczak, K. (2020). AON-induced splice-switching and DMPK pre-mRNA degradation as potential therapeutic approaches for myotonic dystrophy type 1. Nucleic Acids Res. *48*, 2531–2543. https://doi.org/10.1093/nar/gkaa007.

40. Day, J.W., Finkel, R.S., Chiriboga, C.A., Connolly, A.M., Crawford, T.O., Darras, B.T., Iannaccone, S.T., Kuntz, N.L., Peña, L.D.M., Shieh, P.B., et al. (2021). Onasemnogene abeparvovec gene therapy for symptomatic infantile-onset spinal muscular atrophy in patients with two copies of SMN2 (STR1VE): an open-label, single-arm, multicentre, phase 3 trial. Lancet Neurol. *20*, 284–293. https://doi.org/10.1016/S1474-4422(21)00001-6.

41. Belbellaa, B., Reutenauer, L., Messaddeq, N., Monassier, L., and Puccio, H. (2020). High levels of frataxin overexpression lead to mitochondrial and cardiac toxicity in mouse models. Mol. Ther. Methods Clin. Dev. *19*, 120–138. https://doi.org/10.1016/j.omtm.2020.08.018.

42. Yue, Y., Wasala, N.B., Bostick, B., and Duan, D. (2016). 100-fold but not 50-fold dystrophin overexpression aggravates electrocardiographic defects in the mdx model of Duchenne muscular dystrophy. Mol. Ther. Methods Clin. Dev. *3*, 16045. https://doi.org/10.1038/mtm.2016.45.

43. Charizanis, K., Lee, K.Y., Batra, R., Goodwin, M., Zhang, C., Yuan, Y., Shiue, L., Cline, M., Scotti, M.M., Xia, G., et al. (2012). Muscleblind-like 2-mediated alternative splicing in the developing brain and dysregulation in myotonic dystrophy. Neuron *75*, 437–450. https://doi.org/10.1016/j.neuron.2012.05.029.

44. Lin, X., Miller, J.W., Mankodi, A., Kanadia, R.N., Yuan, Y., Moxley, R.T., Swanson, M.S., and Thornton, C.A. (2006). Failure of MBNL1-dependent post-natal splicing transitions in myotonic dystrophy. Hum. Mol. Genet. *15*, 2087–2097. https://doi.org/10.1093/hmg/ddl132.

45. Wagner, S.D., Struck, A.J., Gupta, R., Farnsworth, D.R., Mahady, A.E., Eichinger, K., Thornton, C.A., Wang, E.T., and Berglund, J.A. (2016). Dose-dependent regulation of alternative splicing by MBNL proteins reveals biomarkers for myotonic dystrophy. PLoS Genet. *12*, e1006316–e1006324. https://doi.org/10.1371/journal.pgen.1006316.

46. Sznajder, Ł.J., Michalak, M., Taylor, K., Cywoniuk, P., Kabza, M., Wojtkowiak-Szlachcic, A., Matłoka, M., Konieczny, P., and Sobczak, K. (2016). Mechanistic determinants of MBNL activity. Nucleic Acids Res. *44*, 10326–10342. https://doi.org/10.1093/nar/gkw915.

47. Izzo, M., Battistini, J., Provenzano, C., Martelli, F., Cardinali, B., and Falcone, G. (2022). Molecular therapies for myotonic dystrophy type 1: from small drugs to gene editing. Int. J. Mol. Sci. *23*, 4622. https://doi.org/10.3390/ijms23094622.

48. López-Martínez, A., Soblechero-Martín, P., De-La-puente-ovejero, L., Nogales-Gadea, G., and Arechavala-Gomeza, V. (2020). An overview of alternative splicing defects implicated in myotonic dystrophy type i. Genes *11*, E1109–E1127. https://doi.org/10.3390/genes11091109.

49. Tran, H., Gourrier, N., Lemercier-Neuillet, C., Dhaenens, C.M., Vautrin, A., Fernandez-Gomez, F.J., Arandel, L., Carpentier, C., Obriot, H., Eddarkaoui, S., et al. (2011). Analysis of exonic regions involved in nuclear localization, splicing activity, and dimerization of muscleblind-like-1 isoforms. J. Biol. Chem. *286*, 16435–16446. https://doi.org/10.1074/jbc.M110.194928.

50. Konieczny, P., Stepniak-Konieczna, E., Taylor, K., Sznajder, L.J., and Sobczak, K. (2017). Autoregulation of MBNL1 function by exon 1 exclusion from MBNL1transcript. Nucleic Acids Res. *45*, 1760–1775. https://doi.org/10.1093/nar/gkw1158.

51. Ho, T.H., Savkur, R.S., Poulos, M.G., Mancini, M.A., Swanson, M.S., and Cooper, T.A. (2005). Colocalization of muscleblind with RNA foci is separable from mis-regulation of alternative splicing in myotonic dystrophy. J. Cell Sci. *118*, 2923–2933. https://doi.org/10.1242/jcs.02404.

52. Cywoniuk, P., Taylor, K., Sznajder, Ł.J., and Sobczak, K. (2017). Hybrid splicing minigene and antisense oligonucleotides as efficient tools to determine functional protein/RNA interactions. Sci. Rep. *7*, 17587–17614. https://doi.org/10.1038/s41598-017-17816-x.

OMTN, Volume *30*

Supplemental information

Sustainable recovery of MBNL activity in autoregulatory feedback loop in myotonic dystrophy

Zuzanna Rogalska and Krzysztof Sobczak

Table S1. Primers used for genetic construct preparation.

name of primer	sequence 5` -> 3`
SRCglo_F	CCATTACAACCCGTGCAAGAGGGTAAGGAGT
SRCglo_R	AACTGAAAACATTGGATCTGGAAGGGGAAA
MB22mut_F	TGCTTGCTTGCTTGCTTCCAGTCAGGGTGGGCCG
MB22mut_R	GCAAGCAAGCAAGCAACGGCTCCAGGTGGAGCTGC
MB22del_F	CAAGGTCAGGGCTGCAGTGGGGGGGG
MB22del_R	GCAGCCCTGACCTTGGATGCGACGCG
GFPop_F	TAGGGATCCACCGGATCT
GFPop_R	CTTATCGTCGTCATCCTTGTAATCT
GFPvec_F	GATGACGACGATAAGGTGAGCAAGGGCGAGGAGC
GFPvec_R	TCCGGTGGATCCCTACTTGTACAGCTCGTCCATGCC

Table S2. siRNA sequences

siRNA	sequence 5` -> 3`
**siCtrl sense**	p- UCGAAGUAUUCCGCGUACGdTdT
**siCtrl antisense**	p- CGUACGCGGAAUACUUCGAdTdT
**siMBNL1 sense**	p- GGACGAGGUCAUUAGCCAUdTdT
**siMBNL1 antisense**	p- AUGGCUAAUGACCUCGUCCdTdT

Table S3. PCR primers used for alternative splicing assays and real-time qPCR analysis

PCR target	Primer set	sequence 5` -> 3`
MB22 ex22	pEGFP2_F	ACCGGACTCAGATCTCGAATG
	MBex3_R	ACCAGGCTTGGAGAAACAG
Minigene *Atp2a1* ex22	SrcMins_F	GATCTTCAAGCTCCGGGCCCTG
	SrcMins_R	AGCAATCAGCTAGTCAGTTGCC
Minigene *Nfix* ex7	pDUP51_F	GCAACCTCAAACAGACACCA
	pDUP51_R	AGCTTGTCACAGTGCAGCTC
Minigene *MBNL1* ex5	54ex_F	GCTGCCCAATACCAGGTCAAC
	pcDNA RT-spec_R	AAAGGACAGTGGGAGTGGC
INSR ex11	INSR_F	CCAAAGACAGACTCTCAGAT
	INSR_R	AACATCGCCAAGGGACCTGC
FLNB ex31	FLNB_F	GCTTCGGTGGTGTTGATATTC
	FLNB_R	GTCACTCACTGGGACATAGG
MYO5A ex22	MYO5A_F	GAACAACCGACAGCAGCAG
	MYO5A_R	TTACGGACCGTCTTATCCTG
MBNL2 ex5	MBNL2ex5_F	ATTTTCACCCTCCTGCACAC
	MBNL2ex5_R	CAAGACGCTGGGGTTAAAGA
MBNL2 ex7	MBNL2ex7_F	TCCTTTACCAAAGAGACAAGCAC
	MBNL2ex7_R	CTCAATGCAGATTCTTGGCATTCC
	MBNL1ex1_F	CAGCGACATGCAACAGTCTT

MBNL1 ex1	MBNL1ex1_R	TGTCAGCAGGATGAGCAAAC
NCOR2 ex19	NCOR2_F	ACACCCACAACCGGAATGAGCCTG
	NCOR2_R	GGACTTGGCTTTTCGGCTGCTG
PHKA1 ex19	PHKA1_F	TGCACACACTTGAGCTTCATGGA
	PHKA1_R	AAAGTCCACCTCCCCAGACTGGTC
GAPDH	GAPDH_F	GAGTCAACGGATTTGGTCGT
	GAPDH_R	TTGATTTTGGAGGGATCTCG
	DMPK_F	GCGATCTCTGCCTGCTTACT
DMPK UTR	DMPK_R	GTCCTAGGTGGGGACAGACA

a

gtgatgcggttttggcagtacatcaatgggcgtggatagcggtttgactcacggggatttccaagtctccaccccattgacgtcaatgggagtttgttttggcaccaaaa
tcaacgggactttccaaaatgtcgtaacaactccgccccattgacgcaaatgggcggtaggcgtgtacggtgggaggtctatataagcagagctggtttagtgaaccg
tcagatccgctagcgcaccggactcagatctcgaATGGCTGTTAGTGTCACACCAATTCGGGACACAAAATGGCTAACACTGGAAGTATGTA
GAGAGTTCCAGAGGGGGACTTGCTCACGGCCAGACACGGAATGTAAATTTGCACATCCTTCGAAAAGCTGCCAAGTTGAAA
ATGGACGAGTAATCGCCTGCTTTGATTCATTGAAAGGCCGTTGCTCCAGGGAGAACTGCAAATATCTTCATCCACCCCCACA
TTTAAAAACGCAGTTGGAGATAAATGGACGCAATAACTTGATTCAGCAGAAGAACATGGCCATGTTGGCCCAGCAAATGC
AACTAGCCAATGCCATGATGCCTGGTGCCCCATTACAACCCGTGCAAGAGGgtaaggagtgccctctctgtcccaagccctggccc
caccacagccccttccccatgacgccgcccccgccccgccccgtactttgcaggtggtaagtttctcagccctggcaggacctgtgtccgccccgttccccctgcgcct
gcaggggccacatctccggggcagccccactgcctcctcagcccccacagcccctatagcccccatgccacctccctgccttgataacagtgcctcttgtcctctctggccat
agGATAACTGTTCCCCCTCCTCCATCTCTGAGCCCGTGTCACAGgtatcaccccccttcttgccctcagcccagctgctgtgcccctgccacccg
cgccccctcagccccttgcgcgtcgcatccaaggtcacttgtgctcgcagctccacctggagccgttgccactgctgctgctgcgcttccagtcagggtgggccgctggcctccc
actgggcgtcagtttggctcccaggccctgggcagtgccagcctctgggcccgtctgctgcgctgcgttgcgctggctgtgtgctgggctgtctttgctgtggggctgcagtggggg
ggggcggggtgtctggggacgcaggtgagtaggggagaaactggcagggtggtaagcttctgagcctccaggtaagtgcgtgcctgggagatgcacctgggagggacctcgctg
ccctcctgccgcctgcctcatcccttctctttccccttccagATCCAATGTTTTCAGTTGCACCAAGCTTAGCCACCAATGCATCAGCAGCCGCCTTTAAT
CCCTATCTGGGACCTGTTTCTCCAAGCCTGGTCCCGGCAGAGATCTTGCCGACTGCACCAATGTTGGTTACAGGGAATCCGG
GTGTCCCTGTACCTGCAGCTGCTGCAGCTGCTGCACAGAAATTAATGCGAACAGACAGACTTGAGGTATGTCGAGAGTACCA
ACGTGGCAATTGCAACCGAGGAGAAAATGATTGTCGGTTTGCTCATCCTGCTGACAGCACAATGATTGACACCAATGACA
ACACAGTCACTGTGTGTATGGATTACATCAAAGGGAGATGCTCTCGGGAAAAGTGCAAATACTTTCATCCCCCTGCACATT
TGCAAGCCAAGATCAAGGCTGCCCAATACCAGGTCAACCAGGCTGCAGCTGCACAGGCTGCAGCCACCGCAGCTGC
CATGGGAATTCCTCAAGCTGTACTTCCCCCATTACCAAAGAGGCCTGCTCTTGAAAAAAC
CAACGGTGCCACCGCAGTCTTTAACACTGGTATTTTCCAATACCAACAGGCTCTAGCCAACATGCAGTTACAACAGC
ATACAGCATTTCTCCCACCAGGCTCAATATTGTGCATGACACCCGCTACAAGTGTTGTTCCCATGGTGCACGGTGCTACGCC
AGCCACTGTGTCCGCAGCAACAACATCTGCCACAAGTGTTCCCTTCGCTGCAACAGCCACAGCCAACCAGATACCCATAATATCTGCCGA
ACATCTGACTAGCCACAAGTATGTTACCCAGATGGGAGATTACAAGGATGACGACGATAAGTAG
ggatccaccggatctagataactgatcataatcagccataccacatttgtagaggttttacttgctttaaaaaacctcccacacctccccctgaacctgaaacataaaatgaatgcaattgtt
gttgttaacttgtttattgcagcttataatggttacaaaaaagcaatagcatcacaaatttcacaaataaagcatttttttcactgcattctagttgtggtttgtccaaactcatcaatgtatctta

b

gtgatgcggttttggcagtacatcaatgggcgtggatagcggtttgactcacggggatttccaagtctccaccccattgacgtcaatgggagtttgttttggcaccaaaa
tcaacgggactttccaaaatgtcgtaacaactccgccccattgacgcaaatgggcggtaggcgtgtacggtgggaggtctatataagcagagctggtttagtgaaccg
tcagatccgctagcgcaccggactcagatctcgaATGGCTGTTAGTGTCACACCAATTCGGGACACAAAATGGCTAACACTGGAAGTATGTA
GAGAGTTCCAGAGGGGGACTTGCTCACGGCCAGACACGGAATGTAAATTTGCACATCCTTCGAAAAGCTGCCAAGTTGAAA
ATGGACGAGTAATCGCCTGCTTTGATTCATTGAAAGGCCGTTGCTCCAGGGAGAACTGCAAATATCTTCATCCACCCCCACA
TTTAAAAACGCAGTTGGAGATAAATGGACGCAATAACTTGATTCAGCAGAAGAACATGGCCATGTTGGCCCAGCAAATGC
AACTAGCCAATGCCATGATGCCTGGTGCCCCATTACAACCCGTGCAAGAGGgtaaggagtgccctctctgtcccaagccctggccc
caccacagccccttccccatgacgccgcccccgccccgccccgtactttgcaggtggtaagtttctcagccctggcaggacctgtgtccgccccgttccccctgcgcct
gcaggggccacatctccggggcagccccactgcctcctcagcccccacagcccctatagcccccatgccacctccctgccttgataacagtgcctcttgtcctctctggccat
agGATAACTGTTCCCCCTCCTCCATCTCTGAGCCCGTGTCACAGgtatcaccccccttcttgccctcagcccagctgctgtgcccctgccacccg
cgccccctcagccccttgcgcgtcgcatccaaggtcacttgtgctcgcagctccacctggagccgttgcttgcttgcttgcttccagtcagggtgggccgctggcctccc
actgggcgtcagtttggctcccaggccctgggcagtgccagcctctgggcccgtctgctgcgctgcgttgcgctggctgtgtgctgggctgtctttgctgtggggctgcagtggggg
ggggcggggtgtctggggacgcaggtgagtaggggagaaactggcagggtggtaagcttctgagcctccaggtaagtgcgtgcctgggagatgcacctgggagggacctcgctg
ccctcctgccgcctgcctcatcccttctctttccccttccagATCCAATGTTTTCAGTTGCACCAAGCTTAGCCACCAATGCATCAGCAGCCGCCTTTAAT
CCCTATCTGGGACCTGTTTCTCCAAGCCTGGTCCCGGCAGAGATCTTGCCGACTGCACCAATGTTGGTTACAGGGAATCCGG
GTGTCCCTGTACCTGCAGCTGCTGCAGCTGCTGCACAGAAATTAATGCGAACAGACAGACTTGAGGTATGTCGAGAGTACCA
ACGTGGCAATTGCAACCGAGGAGAAAATGATTGTCGGTTTGCTCATCCTGCTGACAGCACAATGATTGACACCAATGACA
ACACAGTCACTGTGTGTATGGATTACATCAAAGGGAGATGCTCTCGGGAAAAGTGCAAATACTTTCATCCCCCTGCACATT
TGCAAGCCAAGATCAAGGCTGCCCAATACCAGGTCAACCAGGCTGCAGCTGCACAGGCTGCAGCCACCGCAGCTGC
CATGGGAATTCCTCAAGCTGTACTTCCCCCATTACCAAAGAGGCCTGCTCTTGAAAAAAC
CAACGGTGCCACCGCAGTCTTTAACACTGGTATTTTCCAATACCAACAGGCTCTAGCCAACATGCAGTTACAACAGC
ATACAGCATTTCTCCCACCAGGCTCAATATTGTGCATGACACCCGCTACAAGTGTTGTTCCCATGGTGCACGGTGCTACGCC
AGCCACTGTGTCCGCAGCAACAACATCTGCCACAAGTGTTCCCTTCGCTGCAACAGCCACAGCCAACCAGATACCCATAATATCTGCCGA
ACATCTGACTAGCCACAAGTATGTTACCCAGATGGGAGATTACAAGGATGACGACGATAAGTAG
ggatccaccggatctagataactgatcataatcagccataccacatttgtagaggttttacttgctttaaaaaacctcccacacctccccctgaacctgaaacataaaatgaatgcaattgtt
gttgttaacttgtttattgcagcttataatggttacaaaaaagcaatagcatcacaaatttcacaaataaagcatttttttcactgcattctagttgtggtttgtccaaactcatcaatgtatctta

c

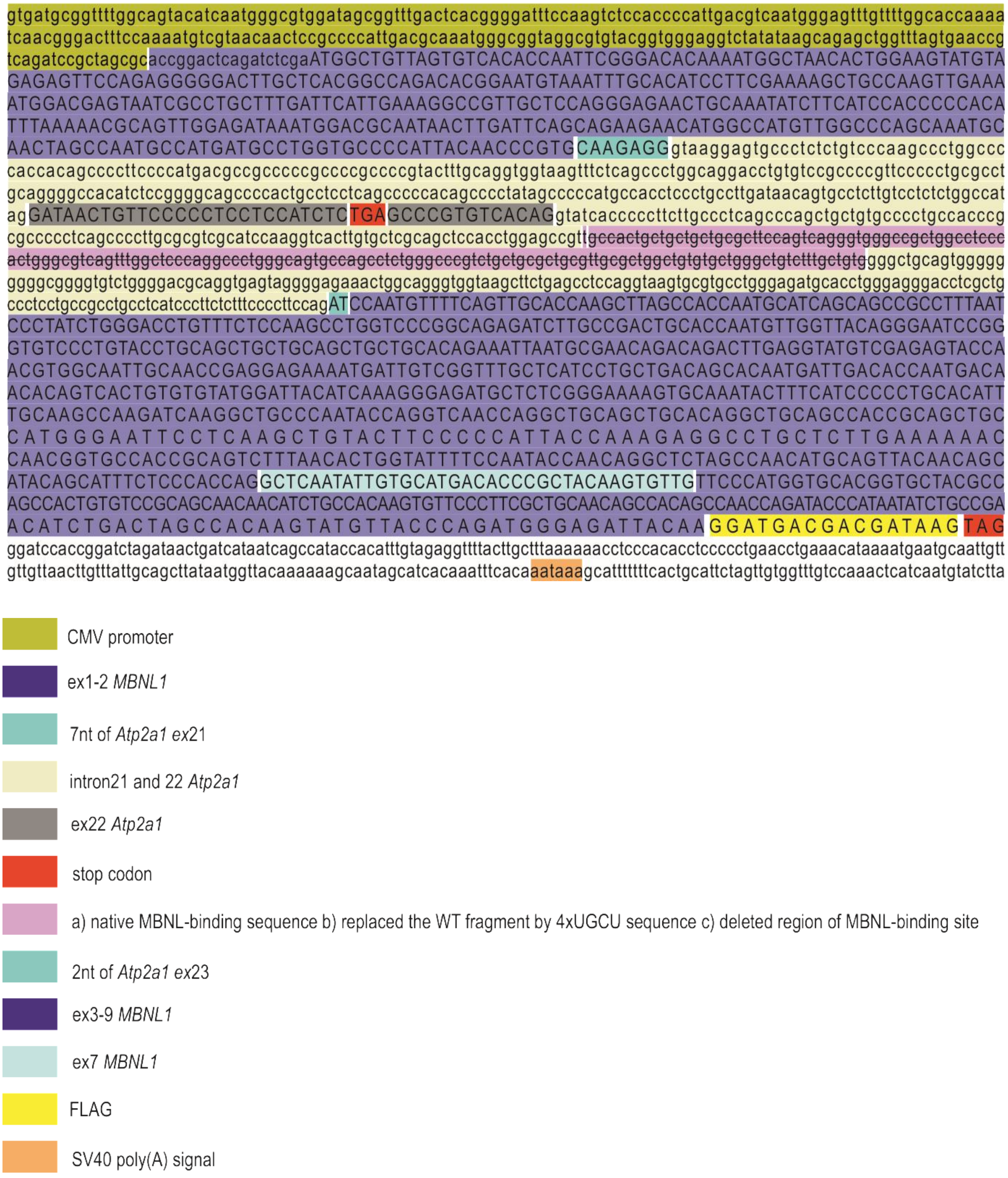

Figure S1. The sequences of MB22 construct with a) native (MB22#1) b) replaced the WT fragment by 4xUGCU sequence (MB22#2) or c) removed MBNL-binding motif (MB22-del) (related to Fig. 1a).

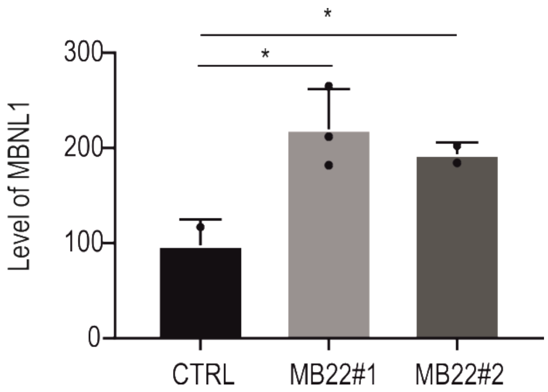

Figure S2. Total level of MBNL1 in MB22 treated cells (related to Fig. 1d).

Results of western blot analysis showing the level of total pool of MBNL1 in cells co-transfected with either MB22#1 or MB22#2 or GFP (CTRL). Anti-MBNL1 antibody staining was carried out. Bars represent average signal from n=3 independent experiments for each group normalized to mCherry. Co-transfection with mCherry expression vector was utilized as a normalization control of transfection. The MB22 treated cells were compared to GFP plasmid treated cells (CTRL); unpaired Student's *t-test*; *$P < 0.05$.

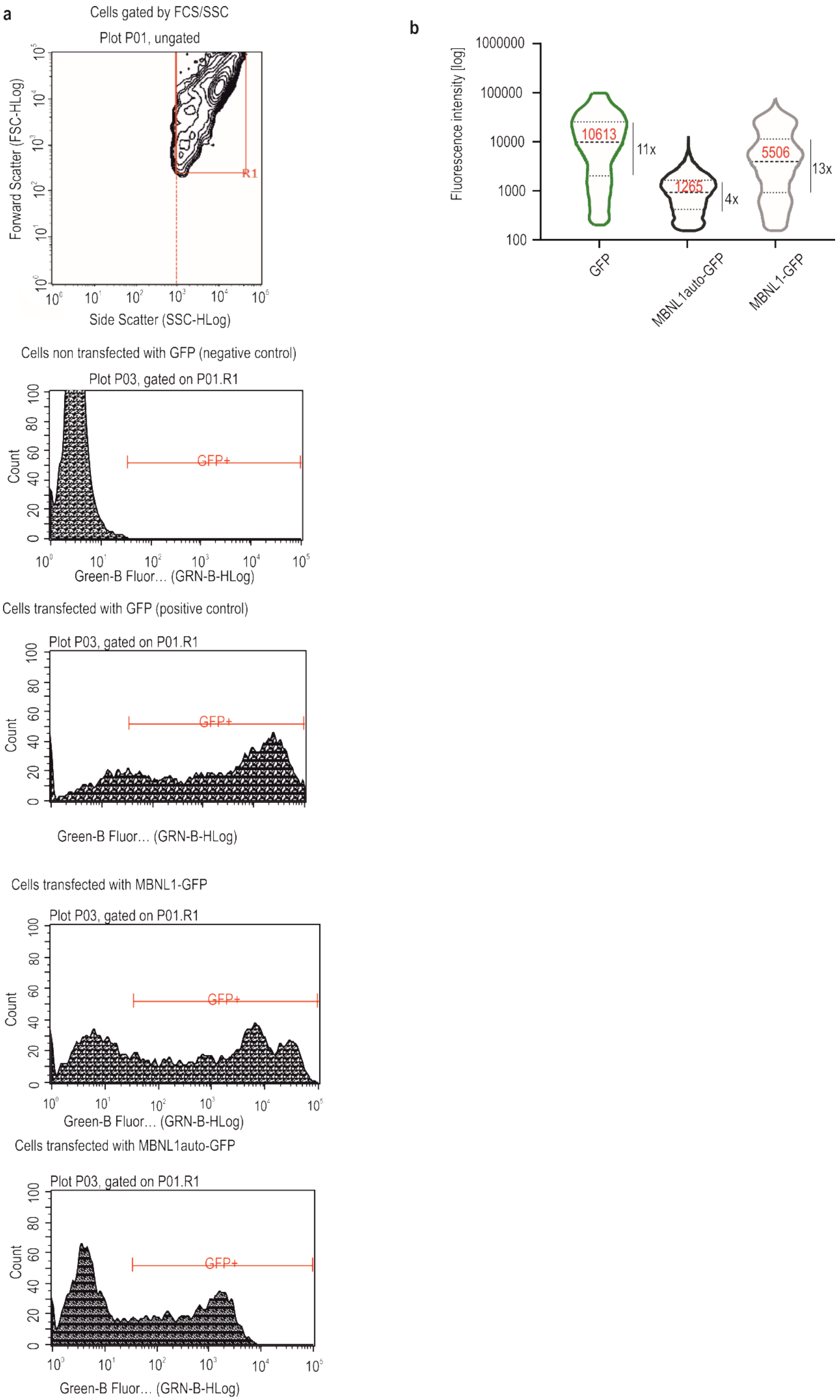
a
Cells gated by FCS/SSC
Plot P01, ungated
Forward Scatter (FSC-HLog)
Side Scatter (SSC-HLog)
R1
Cells non transfected with GFP (negative control)
Plot P03, gated on P01.R1
Count
GFP+
Green-B Fluor... (GRN-B-HLog)
Cells transfected with GFP (positive control)
Plot P03, gated on P01.R1
Count
GFP+
Green-B Fluor... (GRN-B-HLog)
Cells transfected with MBNL1-GFP
Plot P03, gated on P01.R1
Count
GFP+
Green-B Fluor... (GRN-B-HLog)
Cells transfected with MBNL1auto-GFP
Plot P03, gated on P01.R1
Count
GFP+
Green-B Fluor... (GRN-B-HLog)
b
Fluorescence intensity [log]
1000000
100000
10000
1000
100
10613
11x
1265
4x
5506
13x
GFP
MBNL1auto-GFP
MBNL1-GFP

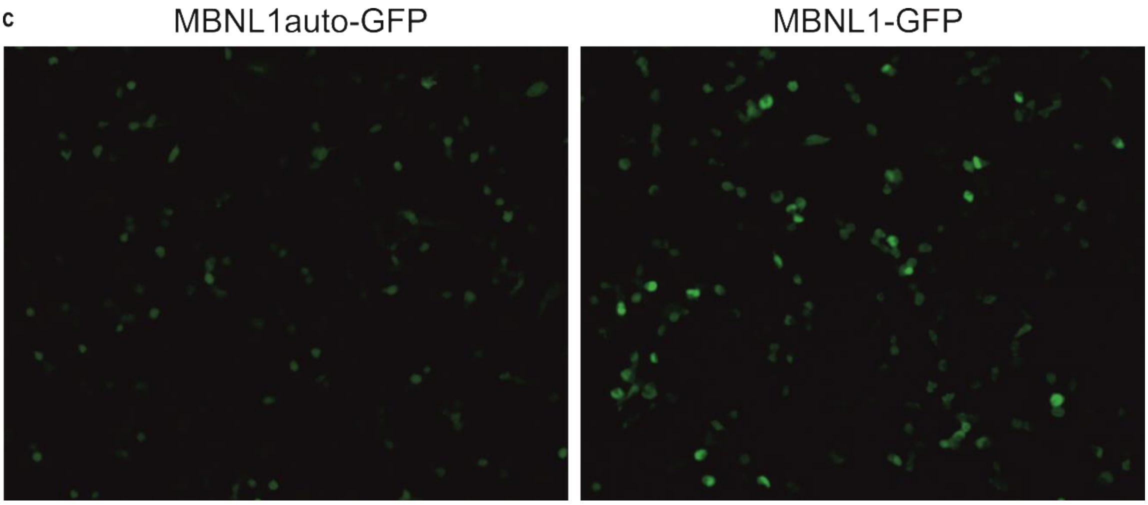

Figure S3. Representative flow cytometry gating and distribution of cells with different fluorescent signal (related to Fig. 3d).

a) For flow cytometry data analysis cells were first gated by Forward Scatter and Side Scatter (FSC/SSC). To distinguish between cells positive and negative for GFP channel, control cell sample (cells non-transfected with GFP referred as mock) were analyzed and utilized as background. Cells transfected with pEGFP-C1 plasmid with strong expression of GFP were also analyzed as positive control. Cells transfected with MBNL1-GFP and MB22#2-GFP represent fluorescence signal from MBNL1-GFP and MBNL1auto-GFP, respectively.

b) Violin plots showing the distribution of cells with different fluorescent signal of either GFP, MBNL1auto-GFP or MBNL1-GFP proteins. COS7 cells were transfected with adequate vectors 48 h prior to flow cytometry analysis. Median fluorescent intensity (black solid line) and 25th and 75th percentile of signal (dashed line) are shown. Fold-change between 25th and 75th percentile of signal for all analyses is also indicated. Cells with signal below 200 were rejected from analyses based on results for control experiment for mock-transfected cells. Signals below 200 were rejected based on analysis of non-transfected cells; Graphs represent values from n=4 independent biological replicates for each experimental condition; N=12916 (GFP), N=6781 (MBNL1auto-GFP) and N=10496 (MBNL1-GFP) cells. The obtained results from GFP, MBNL1-GFP and MBNL1auto-GFP were compared with mock treated as a control; Unpaired Student's *t-test*; *$P < 0.05$; ** $P < 0.01$; *** $P < 0.001$; ns, non-significant.

c) Representative confocal microscopy images showing localization of MBNL1auto-GFP and MBNL1-GFP in COS7 cells transfected with either autoregulated MB22#2-GFP or conventional MBNL1-GFP constructs, respectively.

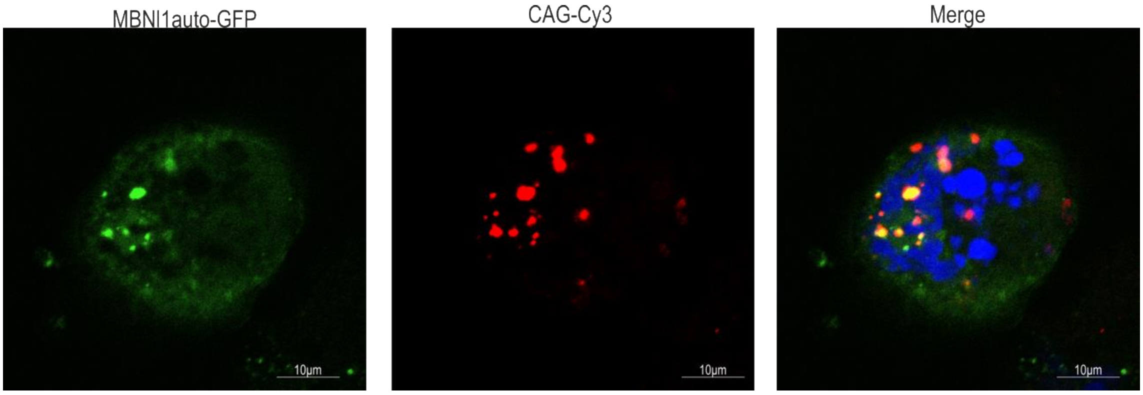

Figure S4. MBNL1auto colocalize with CUG^{exp} in MB22 treated cells (related to Fig. 4a).

Representative confocal images of FISH analysis to visualize the CUG^{exp} nuclear foci containing MBNL1auto in cells co-transfected with plasmid expressing mutant *DMPK* fragment containing CUG_{960} with MB22#2-GFP construct. RNA FISH was performed with DNA/LNA probes $(CAG)_6$-CA labeled at the 5′-end with Cy3 (CAG-Cy3) and MBNL1auto-GFP was visualized with GFP-specific filter (MBNL1auto-GFP); scale bar, 10μm.

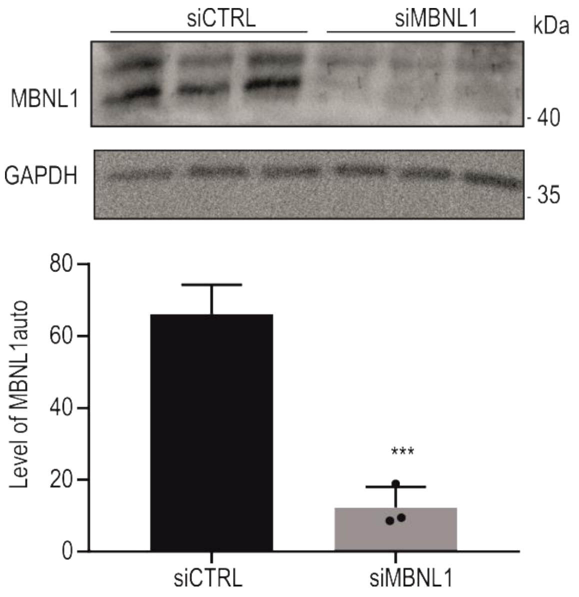

Figure S5. Confirmation of efficient knockdown of *MBNl1* (related to Fig. 4b).

Results of western blot analysis showing the level MBNL1 in COS7 cells treated with si*MBNL1* or siCTRL. Quantification of protein level (anti-MBNL1 antibody) is normalized to GAPDH. The results are averages from n=3 independent experiments; unpaired Student's *t-test*; *** $P < 0.001$ for comparison of siCTRL vs si*MBNL1* groups.

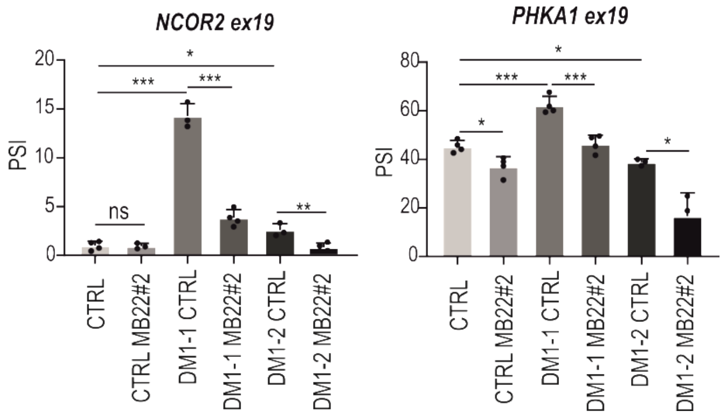

Figure S6. Correction of pathogenic mis-splicing in DM1 cells treated with MB22 lentiviruses (related to Fig. 5b).

Results of RT-PCR analyses showing changes in the regulation of two negatively regulated MBNL-dependent exons. Splicing changes are expressed as PSI. Three different cell line: fibroblast from healthy individuals (non-DM), and two DM1 patients (DM1-1 and DM1-2) were treated with control lentiviral vector (CTRL) or with lentivirus containing MB22#2-GFP sequence. Bars represent average from four independent experiments (dots); unpaired Student's *t-test*; *$P < 0.05$; ** $P < 0.01$; *** $P < 0.001$; ns, non-significant.

Supplementary files

a

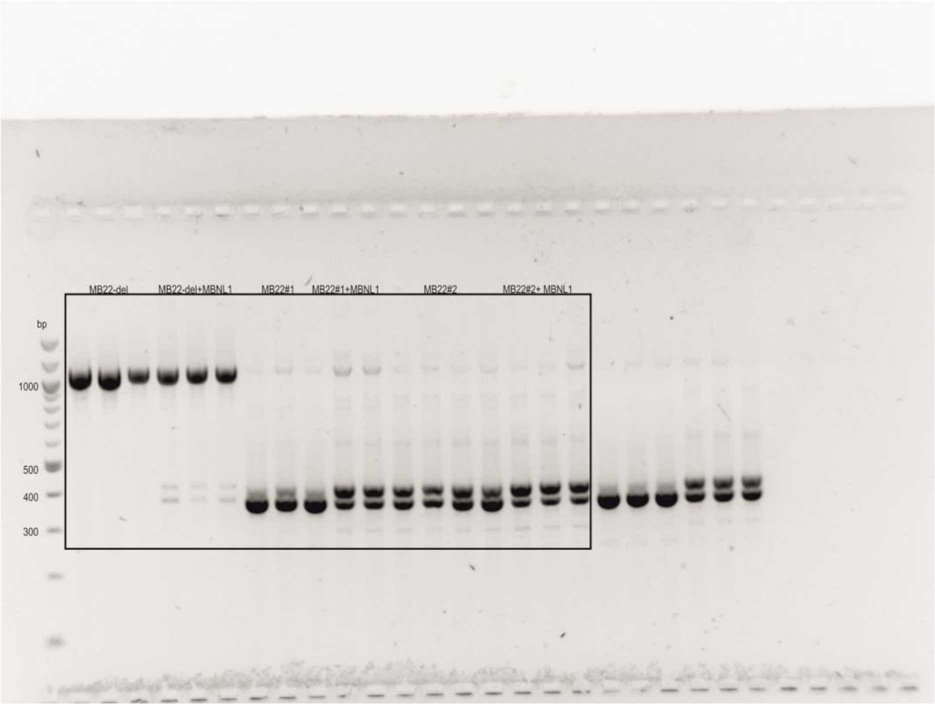

b

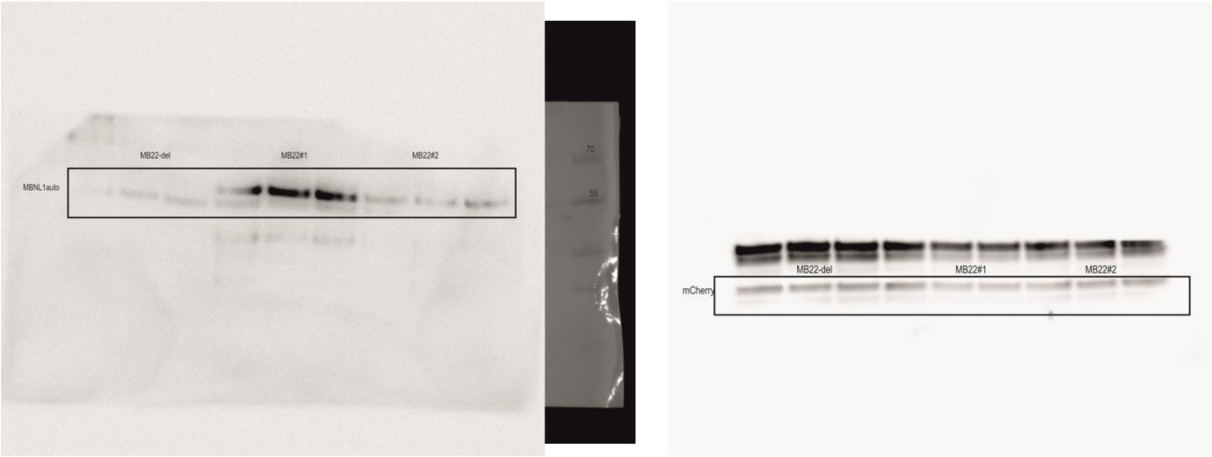

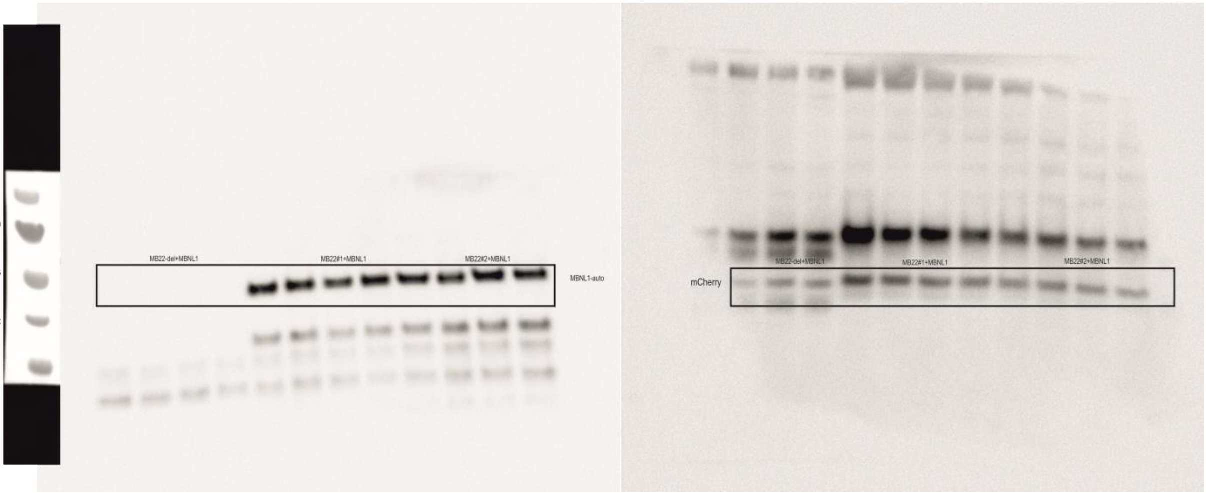

c

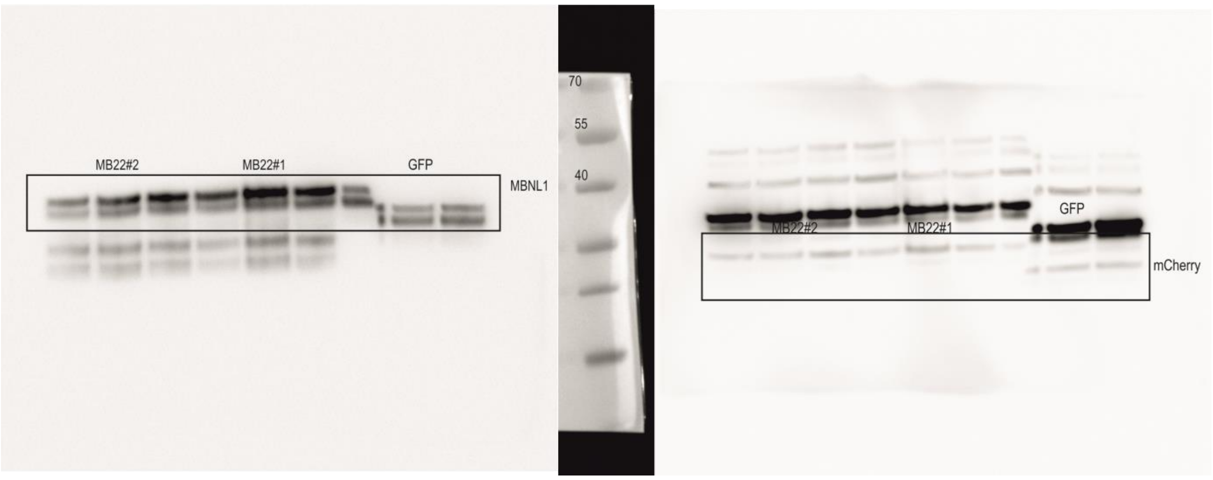

Figure S7 (related to Fig. 1c, d and Fig. S2).

a) Full agarose gel image for RT-PCR products results show in Fig. 1c. Fragments presented in Fig. 1c are indicated by black rectangles. **b)** Full blot image for western blot results show in Fig. 1d. Fragments presented in Fig. 1d are indicated by black rectangles. **c)** Full blot image for western blot results show in Fig. S2. Fragments presented in Fig. S2 are indicated by black rectangles.

COS7

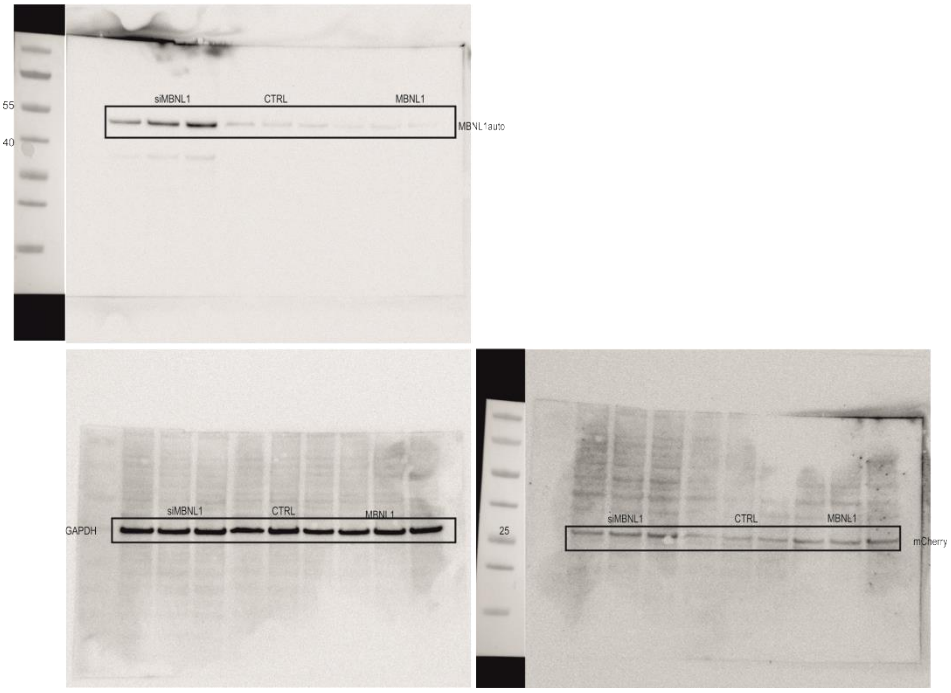
55
40
siMBNL1
CTRL
MBNL1
MBNL1auto
GAPDH
siMBNL1
CTRL
MBNL1
25
siMBNL1
CTRL
MBNL1
mCherry

HEK293

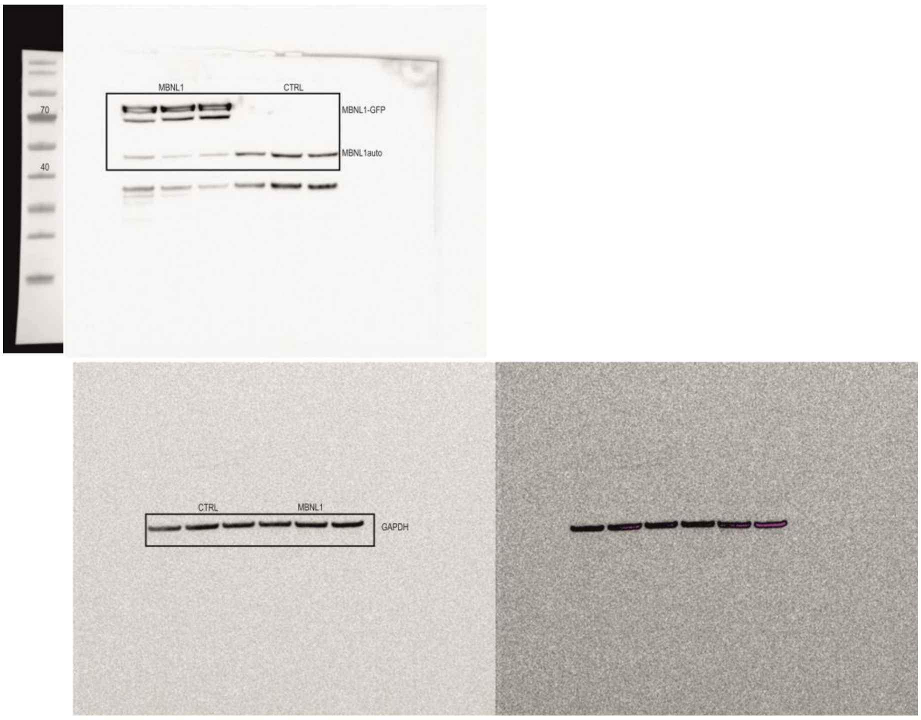
MBNL1
CTRL
70
40
MBNL1-GFP
MBNL1auto
CTRL
MBNL1
GAPDH

MEF 1&2KO

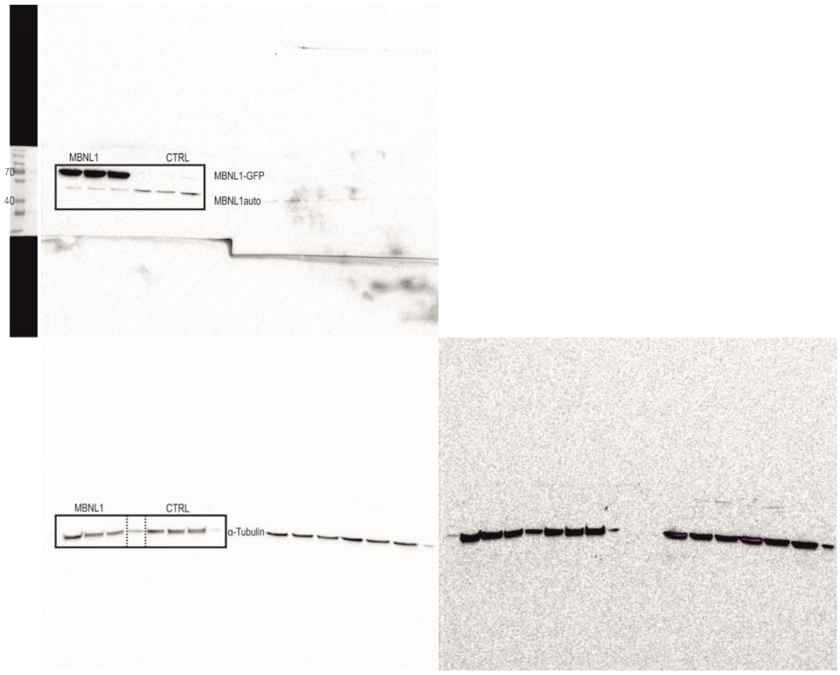

Figure S8 (related to Fig. 2).

Full blot images for western blot results show in Fig. 2. Fragments presented in Fig. 2 are indicated by black rectangles. Additional full blot image with GAPDH for HEK239 and α-Tubulin MEF 1&2KO was added with adjustment of contrast to see blot boundaries.

a

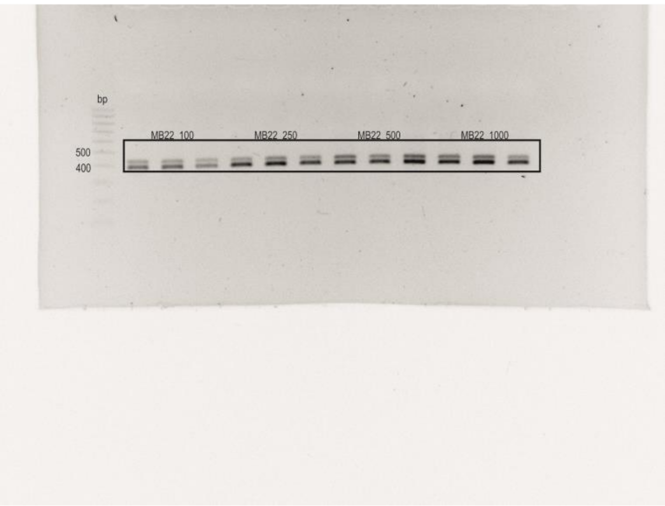

b

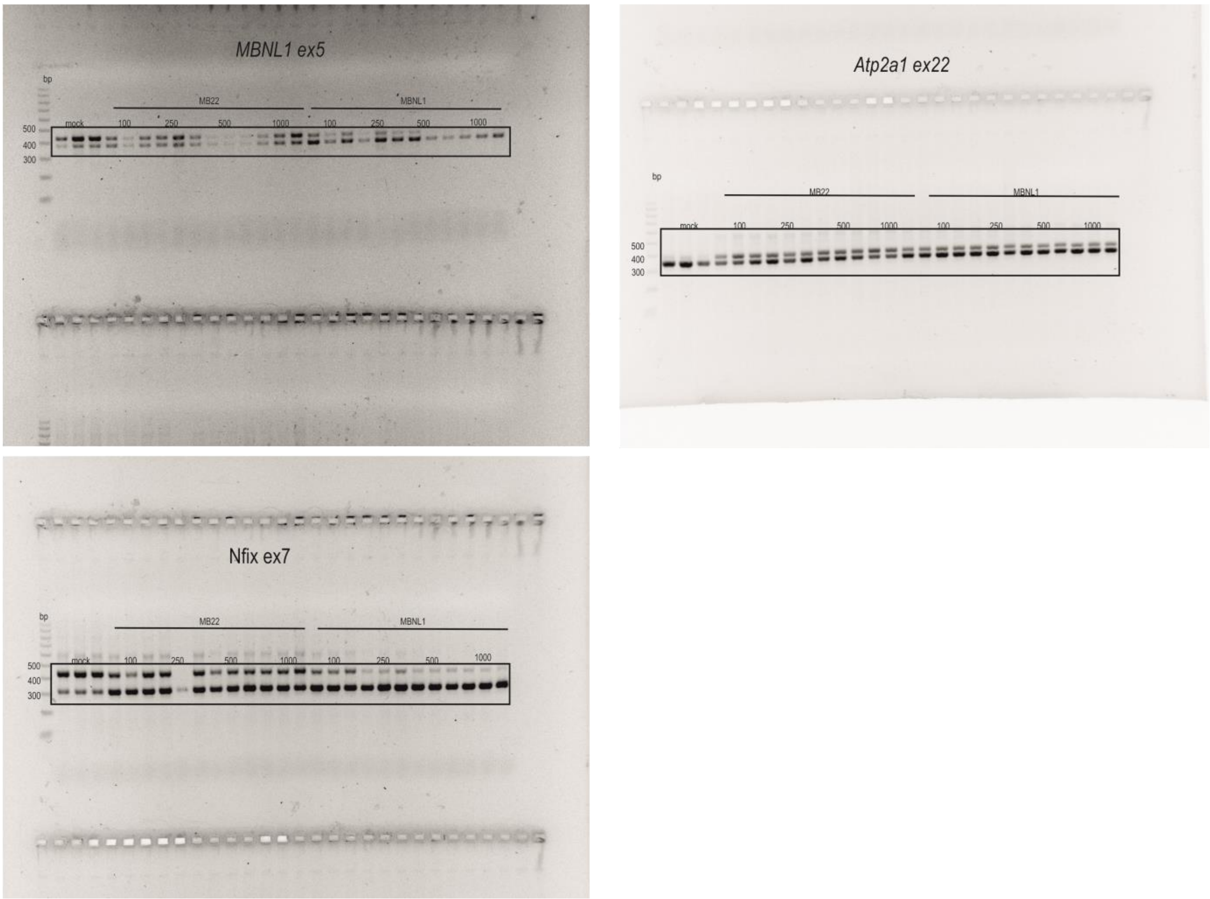

Figure S9 (related to Fig. 3a, b).

a) Full agarose gel image for RT-PCR results show in Fig. 3a. Fragments presented in Fig. 3a are indicated by black rectangles. **b)** Full agarose gel image for RT-PCR results show in Fig. 3b. Fragments presented in Fig. 3b are indicated by black rectangles.

a

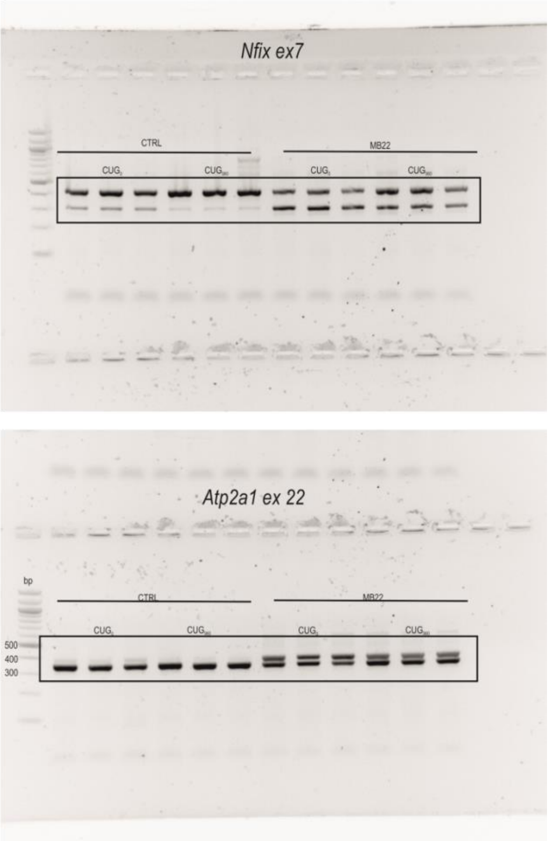

b

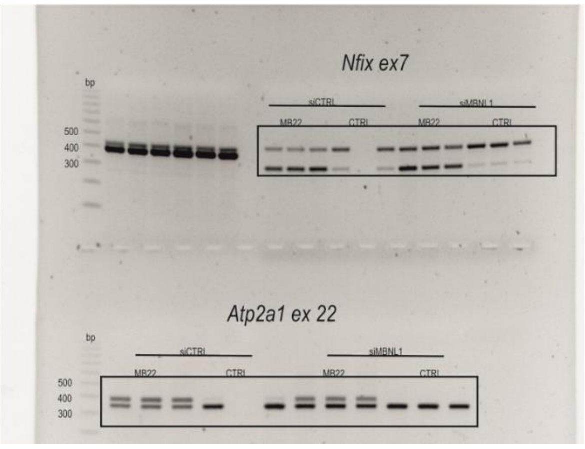

Figure S10 (related to Fig. 4b, c).

a) Full agarose gel image for RT-PCR results show in Fig. 4b. Fragments presented in Fig. 4b are indicated by black rectangles. **b)** Full agarose gel image for RT-PCR results show in Fig. 4c. Fragments presented in Fig. 4c are indicated by black rectangles.

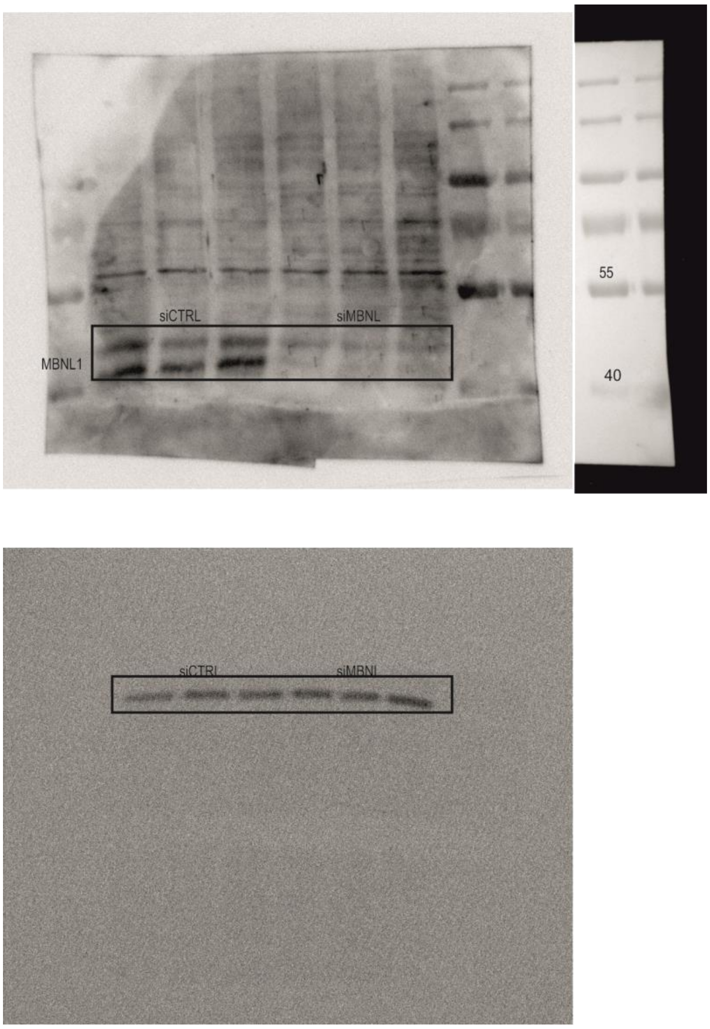

Figure S11 (related to Fig. S5).

Full blot images for western blot results show in Fig. S5. Fragments presented in Fig. S5 are indicated by black rectangles.

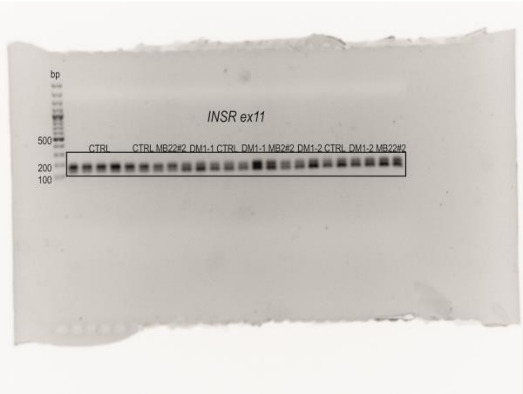
INSR ex11
bp
500
200
100
CTRL CTRL MB22#2 DM1-1 CTRL DM1-1 MB2#2 DM1-2 CTRL DM1-2 MB22#2

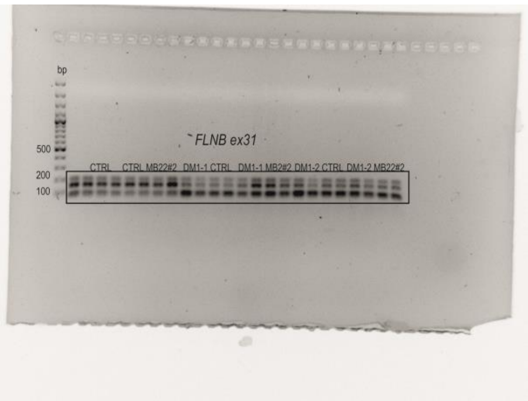
FLNB ex31
bp
500
200
100
CTRL CTRL MB22#2 DM1-1 CTRL DM1-1 MB2#2 DM1-2 CTRL DM1-2 MB22#2

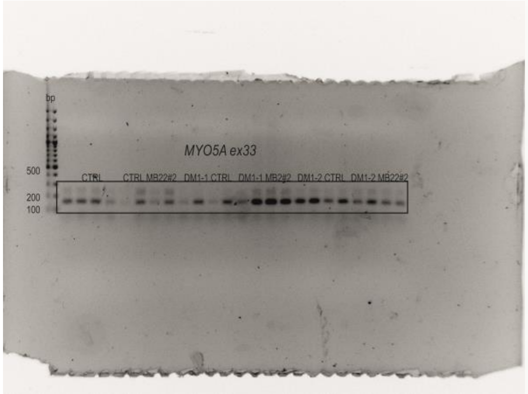
MYO5A ex33
bp
500
200
100
CTRL CTRL MB22#2 DM1-1 CTRL DM1-1 MB2#2 DM1-2 CTRL DM1-2 MB22#2

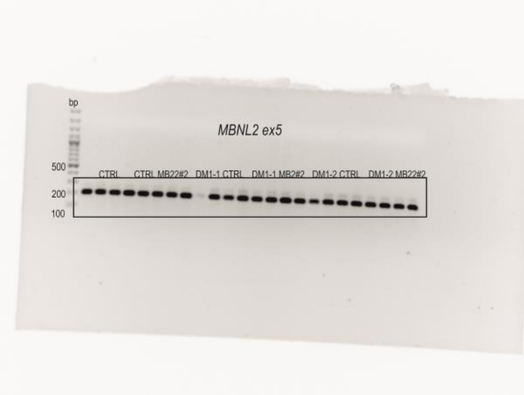
MBNL2 ex5
bp
500
200
100
CTRL CTRL MB22#2 DM1-1 CTRL DM1-1 MB2#2 DM1-2 CTRL DM1-2 MB22#2

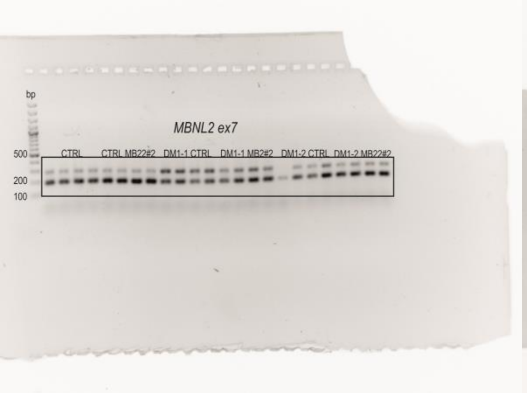
MBNL2 ex7
bp
500
200
100
CTRL CTRL MB22#2 DM1-1 CTRL DM1-1 MB2#2 DM1-2 CTRL DM1-2 MB22#2

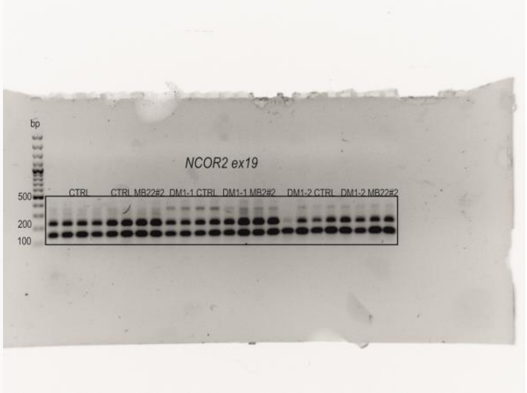
NCOR2 ex19
bp
500
200
100
CTRL CTRL MB22#2 DM1-1 CTRL DM1-1 MB2#2 DM1-2 CTRL DM1-2 MB22#2

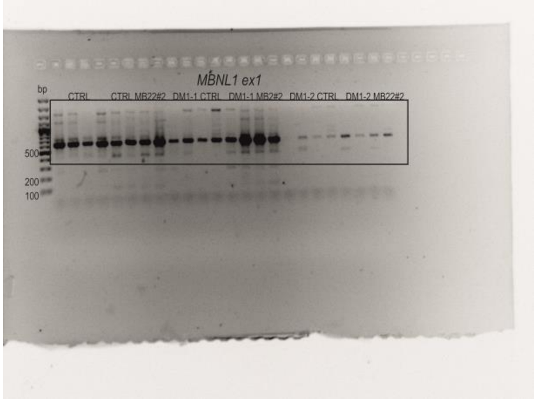
MBNL1 ex1
bp
500
200
100
CTRL CTRL MB22#2 DM1-1 CTRL DM1-1 MB2#2 DM1-2 CTRL DM1-2 MB22#2

Figure S12 (related to Fig. 5b and Fig. S6).

Full agarose gel image for RT-PCR results show in Fig. 5b and Fig. S6. Fragments presented in Fig. 5a and Fig. S6 are indicated by black rectangles.
